# Long-Term Stability of Cord Blood Units After 29 Years of Cryopreservation: Follow-Up Data From the José Carreras Cord Blood Bank

**DOI:** 10.1093/stcltm/szad071

**Published:** 2023-11-04

**Authors:** Stefanie Liedtke, Sabine Többen, Holger Gressmann, Andrea Meyer, Pablo E Verde, Eliane Gluckman, Gesine Kogler

**Affiliations:** Heinrich-Heine-University, Institute for Transplantation Diagnostics and Cell Therapeutics, University Clinic, José Carreras Cord Blood Bank, Düsseldorf, Germany; Heinrich-Heine-University, Institute for Transplantation Diagnostics and Cell Therapeutics, University Clinic, José Carreras Cord Blood Bank, Düsseldorf, Germany; Heinrich-Heine-University, Institute for Transplantation Diagnostics and Cell Therapeutics, University Clinic, José Carreras Cord Blood Bank, Düsseldorf, Germany; Heinrich-Heine-University, Institute for Transplantation Diagnostics and Cell Therapeutics, University Clinic, José Carreras Cord Blood Bank, Düsseldorf, Germany; Heinrich-Heine-University, Coordination Centre for Clinical Trials, University Clinic, Düsseldorf, Germany; Eurocord, Hopital Saint Louis APHP, Institut de Recherche de Saint-Louis (IRSL) EA3518, Université de Paris Cité, Paris, France; Monacord, Centre Scientifique de Monaco, Monaco; Heinrich-Heine-University, Institute for Transplantation Diagnostics and Cell Therapeutics, University Clinic, José Carreras Cord Blood Bank, Düsseldorf, Germany

**Keywords:** cord blood, cryopreservation, transplantation, long-term stability, follow-up data, viability

## Abstract

The José Carreras Cord Blood Bank (CBB) located in Düsseldorf as of today stores 21 215 active cryopreserved cord blood units (CBUs) applicable as a source for hematopoietic stem cell (HSC) transplantation. Since the success of transplantation outcomes is mainly dependent on the cord blood quality, typical parameters are evaluated by a Stability Monitoring Program specified by the FACT Standards. The longest expiration time determined to date is 29 years for unseparated units, 25 years for manual and 18 years for automated volume-reduced units licensed by the Paul-Ehrlich Institute. According to the CBB stability program TNC count, TNC recovery, TNC viability, CD34^+^7AAD^−^ viability, CD45^+^7AAD^−^ viability and CFC count were determined for all 3 processing methods applied over time. As a measure of stability, unseparated units (processed 1993-1998) revealed a mean TNC viability of 88.91 ± 5.01% after 29 years of cryopreservation versus manual volume-reduced CBUs (processed 1998-2005) with a mean of 84.22 ± 10.02% after 25 years of cryopreservation versus automated volume-reduced CBUs (processed since 2005) with a mean of 88.64.91 ± 3.91% after 18 years of cryopreservation. In addition, these relevant parameters were retrospectively analyzed for released transplants in correlation to the storage time. Moreover, the follow-up data of recipients from CBUs cryopreserved directly (unseparated) versus CBUs cryopreserved after manual versus automated volume-reduction are presented here demonstrating an earlier engraftment in both volume-reduced groups as compared to unseparated CBUs. By this retrospective analysis, key questions are discussed regarding cord blood parameters in relation to processing methods, engraftment, and patient age (children and adults).

## Graphical Abstract

**Graphical Abstract:**
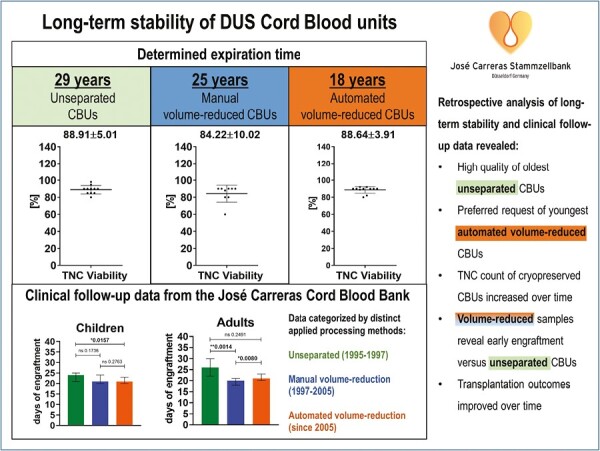

**Significance Statement**

The Cord Blood field needs documented stability evaluations required by both authorities Paul-Ehrlich Institute responsible for licensing and Foundation for the Accreditation of Cellular Therapy. This article comprehensively demonstrates results from the stability program at the Cord Blood Bank Düsseldorf in addition to data from released transplants providing a model for others to test and benchmark the important requirements. Data according to expiration times of 29 years for unseparated, 25 years for manual and 18 years for automated volume-reduced CBUs are presented. Distinct applied processing methods were analyzed retrospectively in released transplants offering a model to test the respective impact of the applied method to required quality.

## Introduction

Cord blood unit (CBU) banking started in Düsseldorf for directed/related donations in 1992 according to the method of Traineau et al.^1^ In 1993, the cryopreservation for allogeneic/unrelated units was established for unseparated units. Since 1996, segments of bags are available for further testing. In December 1997, the manual volume-reduction was introduced according to Rubinstein et al.^2^ Moreover, the closed Sepax100 system was applied for automated volume-reduction as of 2005. The bank is licensed by the local Paul Ehrlich Institute (PEI) since 2001 with an unlimited license since 2010. The CBB achieved the accreditation by the international Foundation for the Accreditation of Cellular Therapy (FACT) in 2004, among the first banks to be accredited. The bank has released 1476 CBUs for allogeneic unrelated/16 CBUs for related transplantation and stores 21 215 active CBUs as of May 2023. Initially, more children were transplanted due to their lower weight and required minimum total nucleated cell (TNC) count per kilogram body weight (TNC ≥ 3.0 × 10^7^/kg). Since 1996, adults were transplanted, although with a lower TNC/kg (TNC ≥ 2.0 × 10^7^/kg).^3-6^ In a recent retrospective study of Kurtzberg et al, which was conducted in collaboration with Eurocord/European Blood and Marrow Transplant Group (EBMT), the largest report of transplantation outcomes in pediatric patients is presented over nearly 30 years.^7^ Here, it is stated that the TNC dose increased significantly over time. Since the beginning of the program, the CBB here has a strong collaboration with Eurocord.^8,9^

In order to ensure the quality of cryopreserved units after long-term storage, a stability monitoring program was established for evaluation of relevant parameters for extension of the expiration date, especially the viability after thawing procedure. In the work of Seo et al, cryopreserved CB samples were analyzed after 1 and 2 years of storage revealing no significant differences for the TNC count, CD34^+^ count and viability.^10^ Lee et al confirmed this data after 5 years of storage, finding as well no significant impact on these parameters.^11^ Moreover, Yamamoto et al extended this analysis for 10 years, showing no difference in the recovery of TNC, CD34^+^ cells, colony-forming units (CFUs), and the viability of CBUs.^12^ However, another work stated a decrease over time for TNC recovery, CD34^+^ count, and viability applying stem cell products obtained from bone marrow (BM), cord blood or mobilized peripheral blood after 11-19 years in the vapor phase of liquid nitrogen.^13^ For cord blood, Broxmeyer et al described an efficient recovery of hematopoietic stem cells (HSC) after ex vivo expansion and mouse NOD/SCID engraftment after 15 years of cryopreservation.^14^ However, these data are derived from thawed aliquots cryopreserved in liquid nitrogen. The same group evaluated already in 2011 the recovery of functional HSC cryopreserved as mononuclear or unseparated cells for 23.5 years in comparison to samples after processing prior to cryopreservation.^15^ As an outcome, CD34^+^ cells revealed a long-term (≥6 months) engrafting capability in immunodeficient mice reflecting the recovery of long-term repopulating, self-renewing HSCs.

The prediction of the engraftment potential is relevant for cellular cord blood products outcome post-transplantation. Currently, there is no single assay, which is accepted in the stem cell field to predict the engraftment potential. The AABB-ISCT Joint Working Group Stability Project Team (SPT) emphasizes the standardization of stability programs for cryopreserved hematopoietic stem/progenitor cells.^16^

Since the CBB bank now has reached the longest validated expiration time of 29 years for unseparated CBUs, 25 years for manual volume-reduced CBUs, and 18 years for automated volume-reduced CBUs, the data presented here provide a detailed analysis correlating to the required extension of the expiration time of product bags stored in liquid nitrogen. Respective data of the CBB internal stability program are analyzed in addition to datasets derived from transplanted CBUs. Moreover, engraftment data are presented in relation to unseparated CBUs versus both volume-reduced groups. This approach should proof the long-term engrafting capability of CBUs stored in liquid nitrogen by analyzing clinical follow-up data and reveal possible storage time-dependent effects on CB quality. Moreover, key questions are discussed about typical CB characteristics in relation to different processing methods, engraftment, and patient age.

## Material and Methods

This study is based on available data from CBB validations, CB inventory database, and follow-up analyses of patients. The retrospective analyses were conducted in cooperation with the EBMT and collaborating transplant centers.

### Stability Program of Cryopreserved CBUs

The stability program includes regulatory requirements specified by the licensing of the PEI and FACT. As of today, the stability program includes in-house validations resulting in regular extension of the expiration time.

### Determination of Expiration Time

To evaluate the expiration time of cryopreserved CBUs, quality parameters like TNC count, TNC recovery after thaw versus prior to cryopreservation, TNC viability, CD34^+^7AAD^−^ viability, CD45^+^7AAD^−^ viability, and total CFC count are determined. At least 7 cryopreserved/thawed bags are to be validated at least 6 months before expiry of the current shelf life. The month of the oldest unit then is reported to the PEI for extension of the expiration time.

### Specification Requirements

Besides mandatory specifications like donor screening/testing, microbial screen, sterility, identity, bag and label integrity, etc., limits are set for parameters tested here according to the current FACT-Netcord standards seventh edition and CBB internal specifications reported to the PEI (Supplementary Table S1).

### Informed Consent

CB collection was carried out in accordance with “The code of Ethics of the World Medical Association” (Declaration of Helsinki) for unrelated cord blood banking with the primary informed consent of the mother according to established criteria.^17,18^ The form includes the voluntary/unpaid donation of CB for the purpose of transplantation of hematopoietic indications but also information on research/discard. The ethical approval was obtained from the ethical review board of the Medical Faculty, University of Duesseldorf (Study No. 2975). The CBB is allowed to contact the mother in case of questions/evaluation of the health status of the child years after donation and data of mother and newborn are pseudonymized by applying a number and letter code.

### Different Processing Methods of CBUs

The allogeneic unrelated CBUs here are cryopreserved in liquid nitrogen (1) either unseparated (in 1992, only related CBUs; since 1993 unrelated CBUs) or volume-reduced applying since 1998, (2) a manual volume-reduction and since 2005, (3) the automated volume-reduction with the Sepax100 system in the presence of Hetastarch in Dextran/DMSO as described previously.^2,17,19^

(1) CBUs with a minimum volume of 60 mL were cryopreserved 1:1 in freezing media containing 78% RPMI1640, 2% human albumin (20%), and 20% DMSO in minimum 2 bags.

(2) Manual volume-reduction was performed by 2 centrifugation steps (step 1: 51g, 5 minutes, 10°; step 2: 400 g, 15 minutes, 10°C) applying a plasma extractor. Applied freezing media consists of 40% DMSO and 60% Dextran (Thomaedex 40, Delta Pharma GmbH).

(3) Automated volume-reduction was performed with the closed automated Sepax 100 system (Cytiva, (former Biosafe), Eysins, Switzerland). DMSO with 40% Dextran (CryoSure-DEX40, WAK Chemie Medical GmbH, Steinbach, Germany) was applied as freezing media.

Freezing of CBUs is performed at a final concentration of 10% DMSO in the Cryoson automatic freezing system BV25 TRA-14 (Consarctic GmbH, Westerngrund, Germany) applied from 1993-2002 or in the Planer Cryosave 32 device applied from 2002 to 2009 or in the Planer Cryosave 560-16 device (both cryotherm GmbH & Co. KG, Euteneuen, Germany) since 2009 with reference electrodes at −1°C per minute and computer-controlled recording. CBUs are transferred to −196°C in liquid nitrogen for quarantine and final storage.

### Thawing/Washing of Cryopreserved CBUs

CBUs are removed from liquid nitrogen (−196°C) and stored for at least 4 hours in the gas phase prior to thawing. Thawing is performed in a sterile overwrap bag (WhirlPAK; neoLab Migge GmbH, Germany) at 37°C in a water bath under agitation. Afterward, the CB bag was installed in the automated Sepax system (Cytiva, (former Biosafe), Eysins, Switzerland) for washing/diluting the cryoprotectant DMSO applying a 1:1 solution of Volulyte 6% (Kabi Pac, Germany; PZN - 2796285) and HSA 5% (Octapharma, Germany).

### Determination of Total Nucleated Cell Count

The determination of TNC counts are performed according to in-house Standard Operating Procedures (SOPs) valid for the respective time frame.

#### Manual Cell Count (1993-1999)

The TNC count was performed by adding 50 µl of blood sample to 950 µl of a 3% acetic acid solution. Afterward, cells were counted on a Neubauer chamber.

#### Automated Cell Count (1999-today)

An automated hematology analyzer (Cell-Dyn Ruby, Abbott Diagnostics) was applied for assessment of TNC count. The number of nucleated cells including erythroblasts is determined by using the mode “CBC+RRBC” (CBC = complete blood count; RRBC = resistant red blood cell). This routine measurement is checked on a workday basis by using Abbott’s quality controls (“Whole blood reference controls CellDynÒ 26 Control”) to ensure accurate cell counts. The TNC recovery is calculated by the following formula: (total cells after thawing/total cells after volume reduction)*100[%].

### Determination of TNC Viability

10 µl of cell suspension is mixed with 10 µl Fuoroquench (OneLambda/BMT Krefeld) and determined under a fluorescent microscope with a 10-fold magnification. The percentage of live leukocytes (green cells) to dead leukocytes (orange/red cells) determines the TNC viability.

### Colony Forming Unit Assay for Determination of Total Colony Forming Cell Count

The presence of precursor cells for the erythroid, myeloid, and megakaryocytic lineage of isolated CD34^+^ cells was determined by culture in a semi-solid medium enriched with hematopoietic growth factors, resulting in red (burst/colony-forming unit erythrocytes [B/CFU-E]), white (colony-forming unit granulocytes/macrophages [CFU-GM]) or mixed colonies (colony-forming unit granulocytes/erythrocytes/macrophages/monocytes [CFU-GEMM]) from the according progenitors. 1 µl of samples prior to cryopreservation and 2.5 µl of thawed samples are seeded in StemMACS HSC-CFU methyl-cellulose medium (Miltenyi, Bergisch Gladbach, Germany) and cultivated at 37°C with 5% CO_2_ in a humidified atmosphere for 14 days. Resulting CFC are counted under a microscope.

### Determination of Viable CD34^+^ Count, CD34^+^7AAD^−^ Viability and CD45^+^7AAD^−^ Leukocyte Viability by Flow Cytometry

100 µl of blood sample are stained with 5 µl of each antibody for 30 minutes in the dark against CD34-PE (clone 8G12; BD Biosciences, Heidelberg, Germany) CD45-FITC (clone 2D1; BD Biosciences, Heidelberg, Germany) and the viability dye 7AAD (Beckman Coulter Inc., France) applying the CytoFlex flow cytometer (Beckman Coulter Inc., France). Using a lyse/no-wash approach for whole-blood samples, 1 mL of Versa Lyse solution (Beckman Coulter Inc., France) is added and incubated for 15 minutes at room temperature in the dark. Determination of CD34^+^ cells was performed according to the guidelines provided by the International Society of Hematotherapy and Graft Engineering (ISHAGE-protocol).^20^

### Statistical Analysis

Data are presented with GraphPad Prism version 8.0.2 as arithmetic means with a standard deviation or as median with 95% confidence interval (CI) for engraftment data. Two-tailed unpaired *t*-tests are conducted to determine significance. A Bonferroni adjustment for a was performed for comparison of 3 categories (processing methods). *P*-values lower than .0167 are considered as significant (*means *P* < .0167; **means *P* < .0033, and ***means *P* < .0003). Linear Regression function was applied to detect possible significant trends. The slopes of the best-fit values are presented with the respective 95% CI and *P*-values.

### Engraftment Data Definition

The date of engraftment is considered as the first of 3 consecutive days of neutrophils ≥ 5 × 10e8/L, without evidence of autologous reconstitution and the date of platelets recovery is considered as the first day platelets ≥ 20 × 10e9/L without platelets transfusion during 7 consecutive days and without evidence of autologous reconstitution or graft rejection in the first 100 days.

## Results

### Stability of Unseparated CBUs After 29 Years of Storage

**Figure 1.**
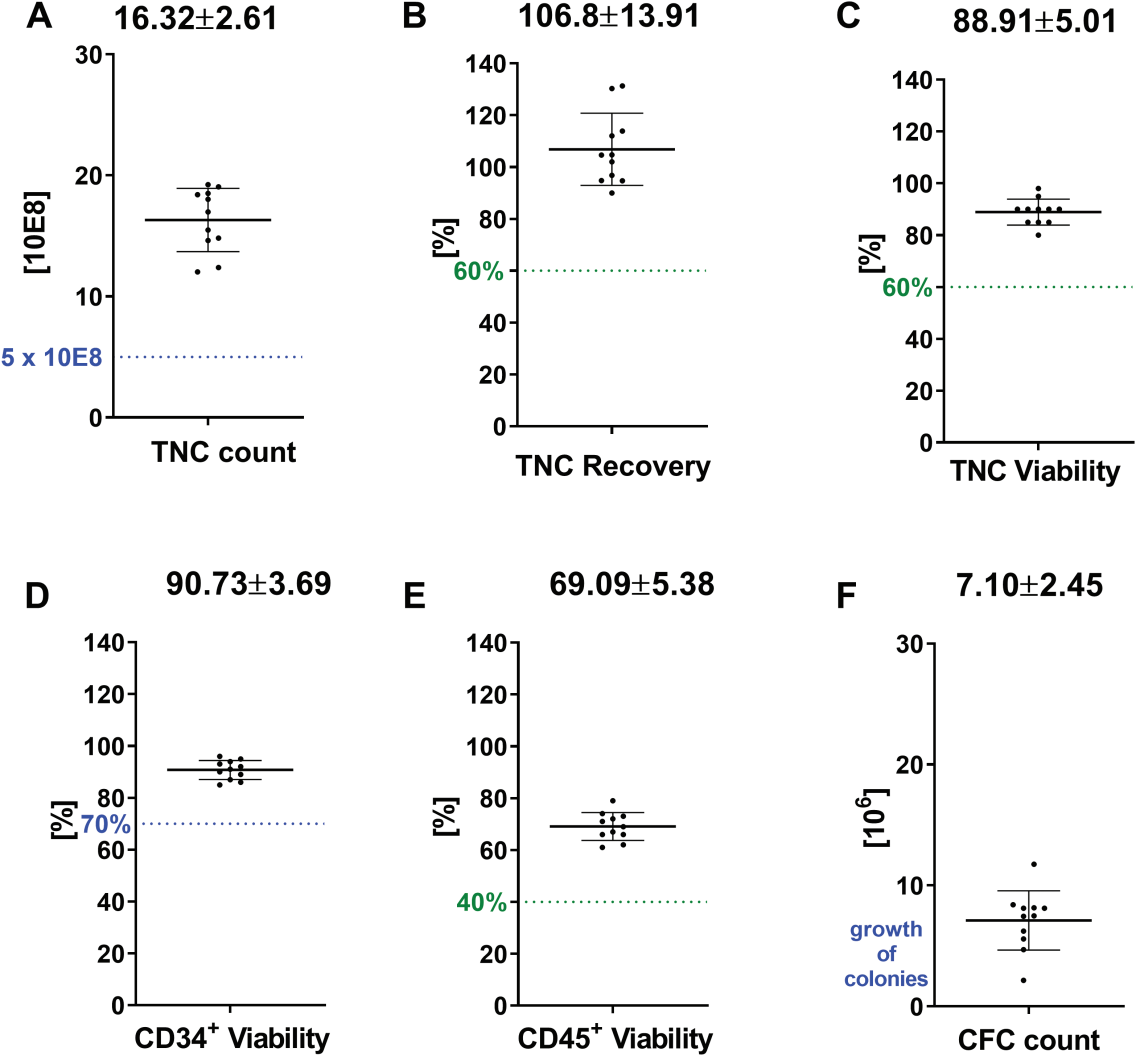
Stability data from unseparated CBUs after 29 years of storage In total, *n* = 11 samples are analyzed after thaw for: (**A**) TNC count, (**B**) TNC recovery, (**C**) TNC viability, (**D**) CD34+ viability, (**E**) CD45+ viability, and (**F**) CFC count. The spotted lines within the graphs depict specific requirements (limits) either requested by FACT (blue) or from internal specifications (green). Means with SD are given above the data.

Results according to the expiration time are presented in Fig. 1. All parameters tested for unseparated CBUs fulfill the specific requirements (Supplementary Table S1). The TNC count reveals a high mean of 16.32 ± 2.61 × 10^8^ correlating to the high individual TNC content in preselected units (Fig. 1A). The TNC recovery has a high mean of 106.8%, maybe resulting from distinct applied cell counting methods (Fig. 1B). Viabilities are very high with a mean TNC viability of 88.91 ± 5.01%, a mean CD34^+^ viability of 90.73 ± 3.69%, and a mean CD45^+^ viability of 69.09 ± 5.38% (Fig. 1C-1E, respectively). The CFC count (Fig. 1F) results in a high mean of 7.10 ± 2.45 × 10^6^ according to a high CD34^+^ count (raw data tables containing additional information for CD34^+^ count, bag volume, transport duration, etc., are given in Supplementary Table S2A). The results of this analysis reflect the high quality of unseparated cryopreserved units after 29 years of storage.

### Stability of Manual Volume-Reduced CBUs After 25 Years of Storage and Correlation With Cryopreservation Time

**Figure 2.**
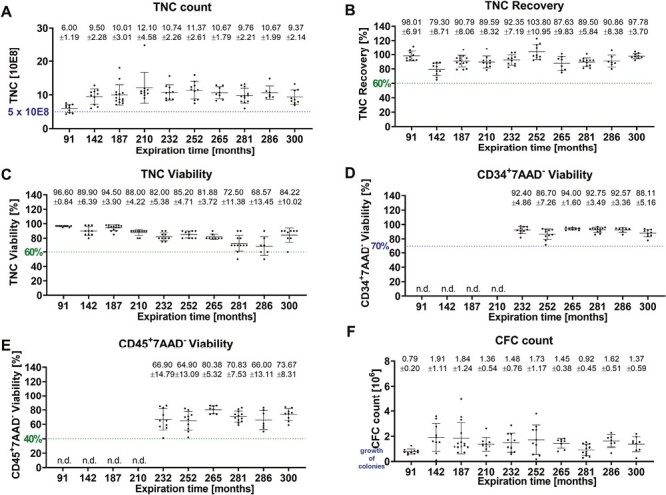
Stability data from manual volume-reduced CBUs after 25 years of storage and correlation with cryopreservation time representative data are shown up to a storage time of 25 years for: (**A**) TNC count, (**B**) TNC recovery, (**C**) TNC viability, (**D**) CD34+ viability, (**E**) CD45+ viability, and (**F**) CFC count. The spotted lines within the graphs depict specific requirements (limits) either requested by FACT (blue) or from internal specifications (green). Means with SD are given above the data.

The extension of the expiration time was performed over time for the licensed volume-reduced product (Fig. 2). The TNC counts (Fig. 2A) ranged between 6.00 ± 1.19 and 12.10 ± 4.58 × 10^8^, and TNC recoveries (Fig. 2B) between 79.30 ± 8.71% and 103.8 ± 10.95%. The TNC viability (Fig. 2C) ranges from 68.57 ± 13.45% to 96.60 ± 0.84. For the TNC viability a slight negative trend is observed after 281 and 286 months, since the means decreased. For the longest expiration time of 300 month the TNC viability resulted in a high mean of 84.22 ± 10.02%. The CD34^+^7AAD^-^ alive HSC reveal a stable viability ranging between 86.70 ± 7.26% and 94.00 ± 1.60% (Fig. 2D). No negative trend is observed for this parameter. In contrast, the leukocyte viability CD45^+^7AAD^−^ (Fig. 2E) shows as expected lower means ranging from 66.00 ± 13.11% up to 80.38 ± 5.32%. Mean values from total CFC counts range from 0.79 ± 0.20 × 10^6^ up to 1.73 ± 1.17 × 10^6^ cells due to individual CFC counts. Here, a storage up to 25 years (300 months) is presented for manual volume-reduced CBUs. No obvious trend related to the storage time was observed except for the TNC viability demonstrating a wider distribution after 286 months (23.8 years).

### Stability of Automated Volume-Reduced CBUs After 18 Years of Storage

**Figure 3.**
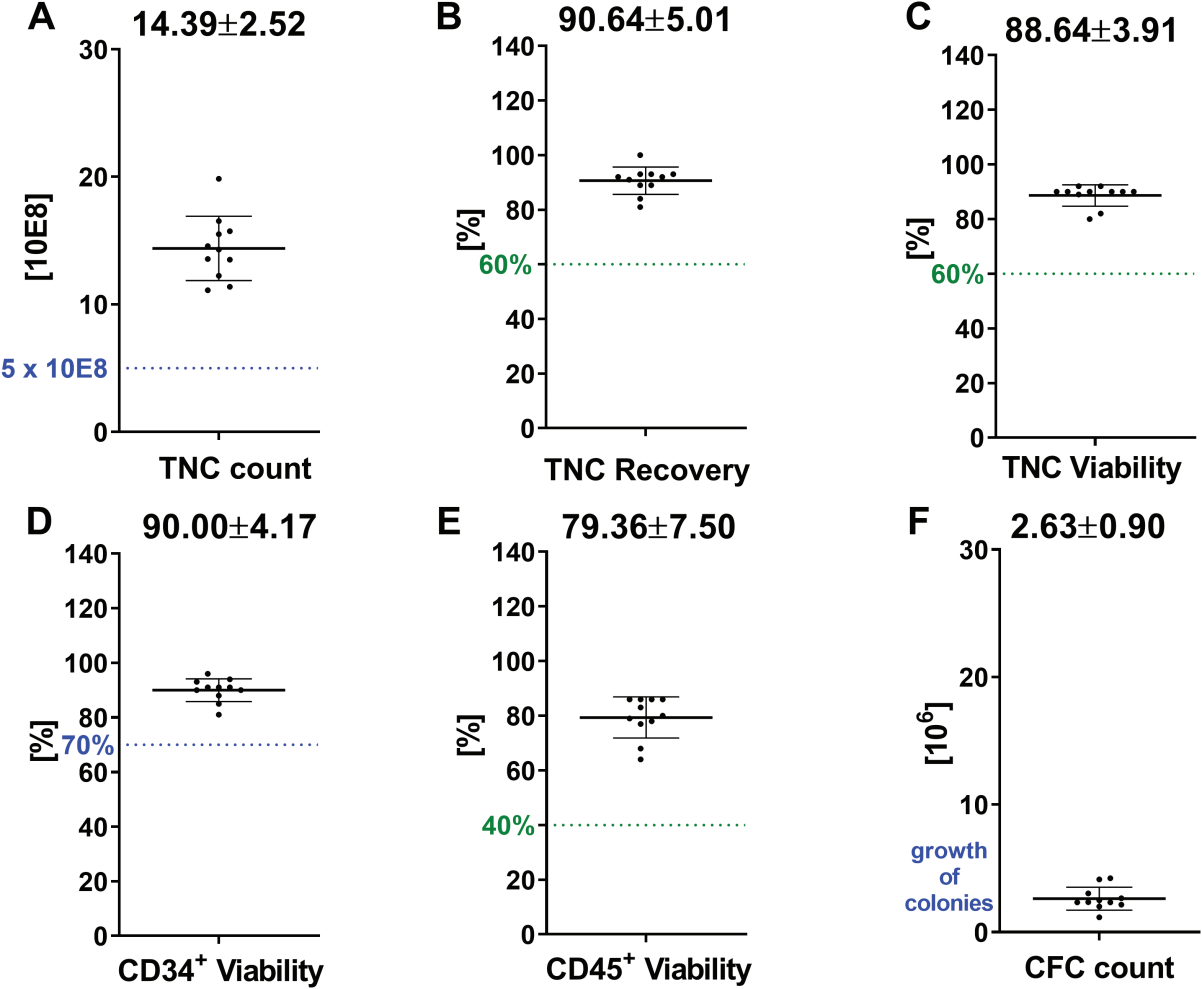
Stability data from automated volume-reduced CBUs after 18 years of storage In total, *n* = 11 samples are analyzed after thaw for: (**A**) TNC count, (**B**) TNC recovery, (**C**) TNC viability, (**D**) CD34+ viability, (**E**) CD45+ viability, and (**F**) CFC count. The spotted lines within the graphs depict specific requirements (limits) either requested by FACT (blue) or from internal specifications (green). Means with SD are given above the data.

The longest cryopreservation time of 18 years (211 months) for automated volume-reduced CBUs was recently tested. Here, the mean TNC count was 14.39 ± 2.52 resulting in a mean TNC recovery of 90.64 ± 5.01% (Fig. 3). TNC viability was 88.64 ± 3.91% as compared to the CD34^+^7AAD^−^ viability with 90.00 ± 4.17% and the CD45^+^7AAD^−^ viability with 79.36 ± 7.50%. CFC count resulted in a mean of 2.63 ± 0.90% confirming a high quality of automated volume-reduced CBUs after 18 years of cryopreservation in liquid nitrogen.

### Trends From CB Inventory

**Figure 4.**
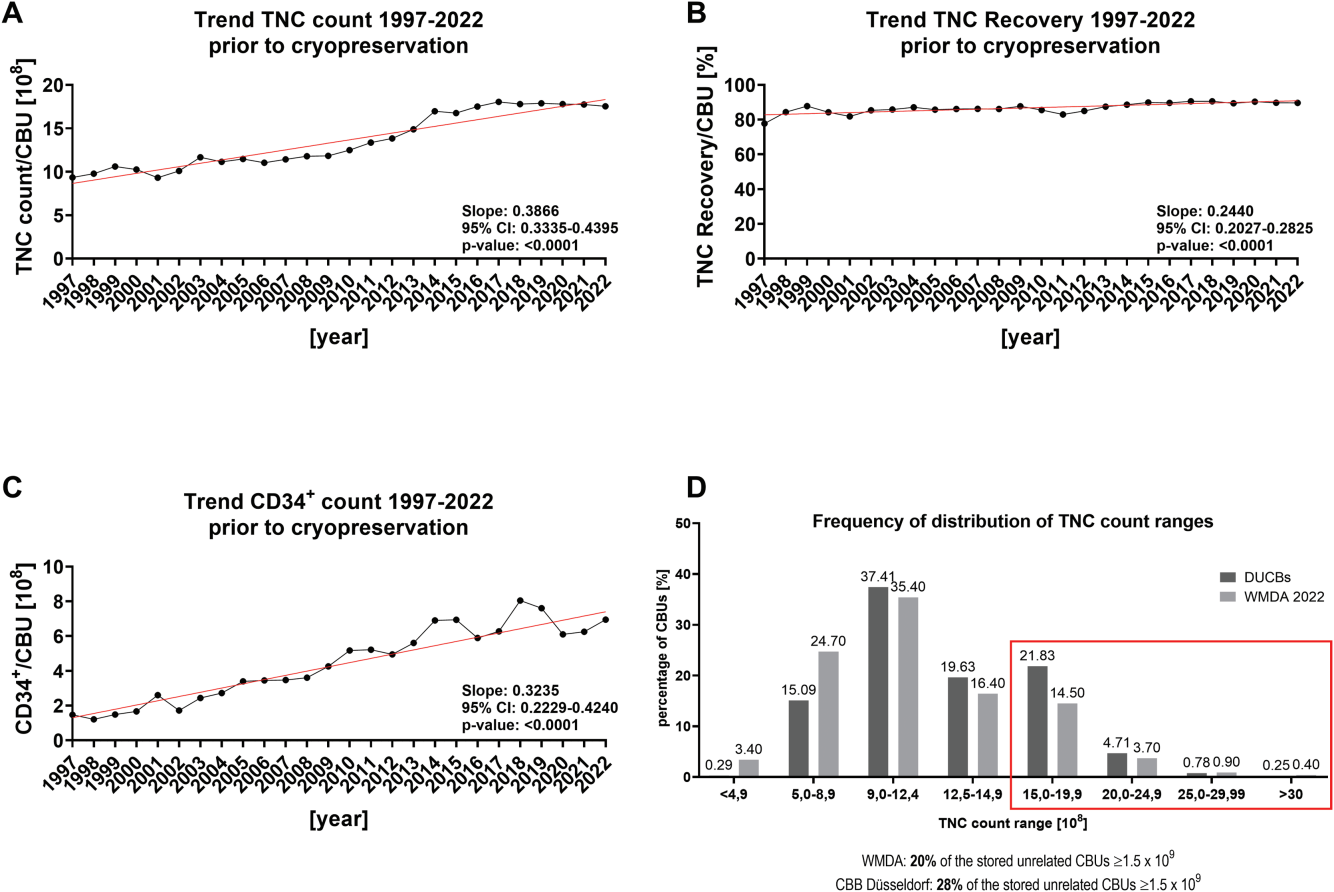
Trends and TNC count ranges of CBB Düsseldorf compared to WMDA data Trends of CB collection are presented from 1997-2022 for TNC count (**A**), TNC recovery (**B**), and CD34+ count (**C**) after processing prior to cryopreservation. Linear regression was applied to visualize possible trends. (**D**) A frequency of distribution is presented for the TNC count ranges after processing prior to cryopreservation for the inventory of the CBB here containing all listed unrelated DUCBs (*n* = 21 042) as of February 2023 versus *n* = 804 832 CBUs provided by the WMDA as of 2022 (https://wmda.info/).

Trends of TNC count, TNC recovery and CD34^+^ count are presented from 1997-2022. Moreover, a detailed analysis of TNC count ranges is presented, as one of the most relevant parameters for transplantation (Fig. 4).

The TNC count significantly increased over time (Fig. 4A). The TNC recovery increased with a positive slope of 0.2440 over time (Fig. 4B). The CD34^+^ count revealed a significant increase, confirming qualified processing methods applied (Fig. 4C).

A peak of TNC counts is demonstrated within the range of 0.9-12.4 × 10^8^ for the CBB inventory here with 37.41% of Düsseldorf CBUs and with 35.40% of the CBUs analyzed by the WMDA (Fig. 4D). 28% of the stored Düsseldorf CBUs within the CBB inventory and 20% of the CBUs analyzed by the WMDA are above a TNC count of ≥ 1.5 × 10^9^. Therefore, the TNC counts of banked CBUs from the CBB inventory here are higher as compared to the data provided by the WMDA confirming the high quality of cryopreserved units.

### Post-Thaw Analysis of Released Transplants According to Distinct Processing Methods

**Figure 5.**
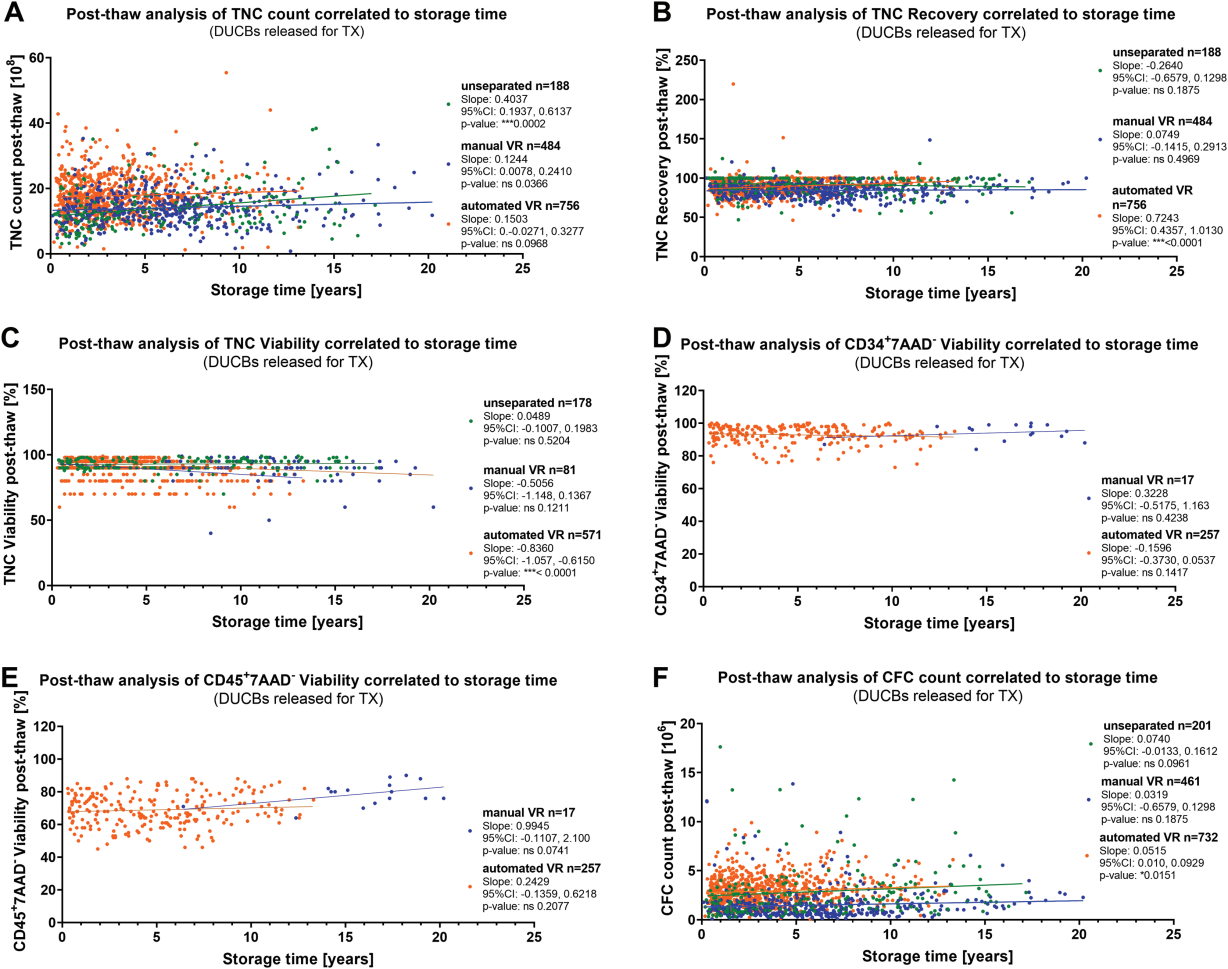
Post-thaw analysis of released transplants correlated to the storage time (**A**) TNC count, (**B**) TNC recovery, (**C**) TNC viability, (**D**) CD34+ viability, (**E**) CD45+ viability, (**F**) CFC count. Data deriving from CBUs processed either unseparated (green) or by manual volume-reduction (blue) and CBUs processed by automated volume-reduction (orange), in order to recognize possible impact of the respective processing method. Lines represent the linear regression of respective datasets with slopes, 95% confidence interval (CI) and *P*-value given in the legend.

In Fig. 5, data from released transplants are plotted against the storage time. The TNC count increased significantly with a ***P-value of <.0001 related to longer storage time in unseparated units; however, both volume-reduced methods show a positive trend (Fig. 5A). For the TNC recovery, no significance was detected (Fig. 5B). In Fig. 5C, significance with a ***P-value of <.0001 was determined in line with the decrease of TNC viability with longer storage time in automated volume-reduced samples. For the CD34^+^7AAD^−^ viability (Fig. 5D), the CD45^+^7AAD^−^ viability (Fig. 5E) and the CFC count (Fig. 5F) no significant trend related to the storage time was observed. Related means (min, max, range, SD) are given in Supplementary Table S3.

In Supplementary Fig. S1A-S1C, frequencies of distributions are given demonstrating TNC count ranges of released transplants represented for the distinct processing methods. Here, it is clearly visible that automated volume-reduced units have higher TNC counts at release for transplantation. 64.59% of these CBUs had a high TNC count of ≥ 15 × 10^8^. Supplementary Fig. S1D shows 24% (48/192) of the unseparated CBUs are made available for transplantation after 0-2 years. The frequency of distribution according to manual volume-reduction revealed with about 22% (105/470) the highest release of transplants after a storage time of ≥ 6-8 years. After that time point, a continuous decrease of released transplants with longer storage was observed (Supplementary Fig. S1E). Approximately 34% (246/731) of the transplants after automated volume-reduction are released after 2 years of storage (Supplementary Fig. S1F).

**Figure 6.**
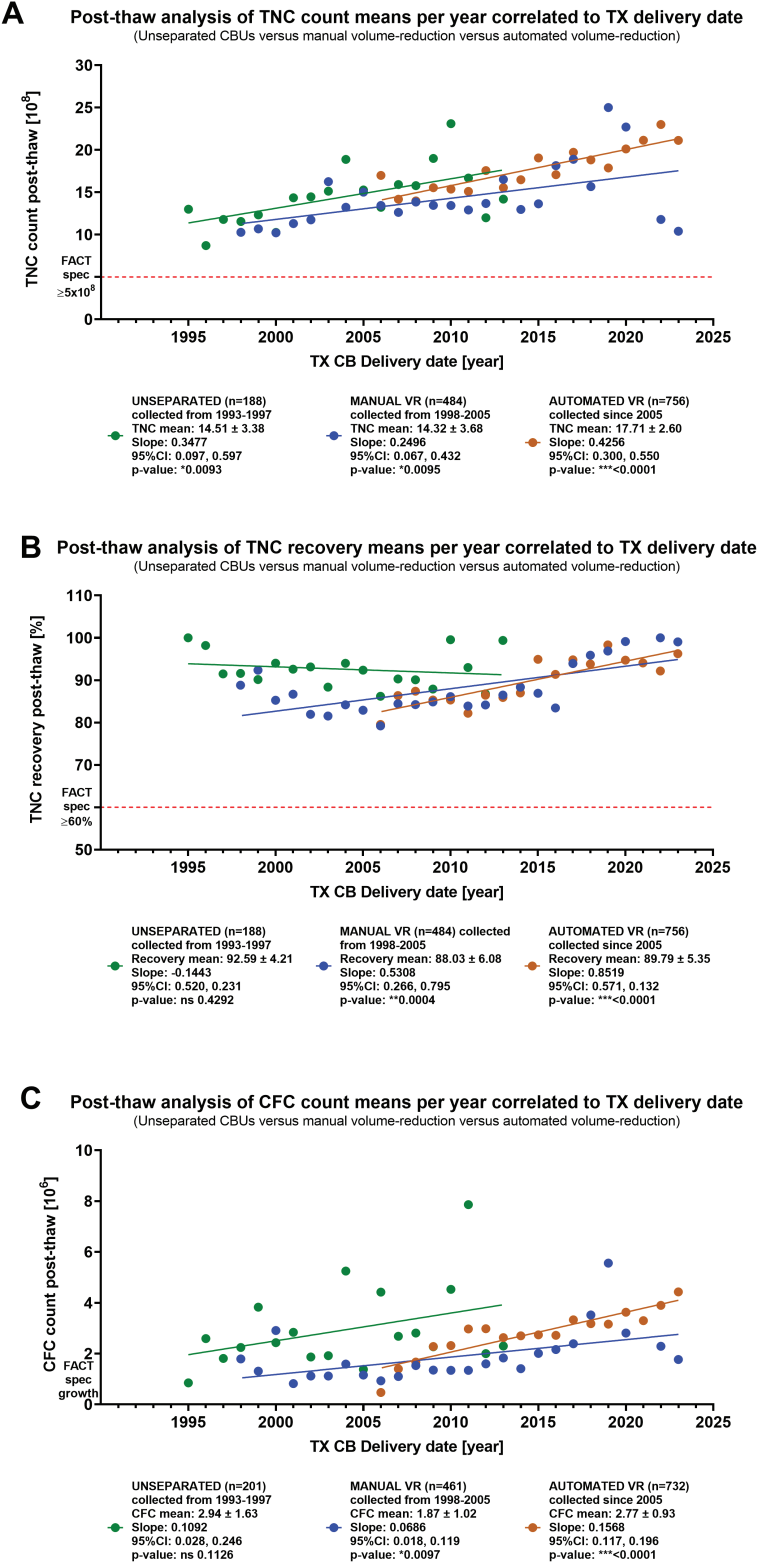
Post-thaw analysis of released transplants for relevant parameters after thaw means per year are correlated to TX delivery date for unseparated CBUs (green dots) versus manual volume-reduced CBUs (blue dots) versus automated volume-reduced CBUs (orange dots) for (**A**) TNC count, (**B**) TNC recovery, and (**C**) CFC count. Dashed red lines mark the FACT specification limit given at the y-axis. Legends beyond the graphs display the mean of all available datapoints for the respective processing method with SD. Lines represent the linear regression of respective datasets.

This preferred request of younger units is in accordance to higher TNC counts over time (Fig. 6A). Each processing method revealed an increase represented by positive slopes of the TNC count. In line with this, the highest TNC count was determined for the automated volume-reduced CBUs with 17.71 ± 2.60 × 10^8^, as compared to 14.32 ± 3.68 × 10^8^ for manual volume-reduced CBUs and 14.51 ± 3.38 × 10^8^ for unseparated CBUs. The TNC recovery was highest in unseparated CBUs with 92.59 ± 4.21% (Fig. 6B) and a negative slope. Slightly lower means are determined for manual volume-reduced CBUs with 88.03 ± 6.06 and automated volume-reduced CBUs with 89.79 ± 5.35%. The highest slope in line with the highest significant increase are observed for automated volume-reduced CBUs. Fig. 6C displays the results for CFC count demonstrating high SDs in between the individual values. A mean of 2.94 ± 1.63 × 10^6^ was determined in unseparated CBUs. In the manual, volume-reduced CBUs a mean of 1.87 ± 1.02 × 10^6^ was determined with a significant increase. A mean of 2.77 ± 0.93 × 10^6^ was determined in the automated volume-reduced samples with the highest slope of 0.1568. In summary, the highest improvement over time was observed in automated volume-reduced CBUs.

### Follow-Up Engraftment Data From Transplanted CBUs

**Figure 7.**
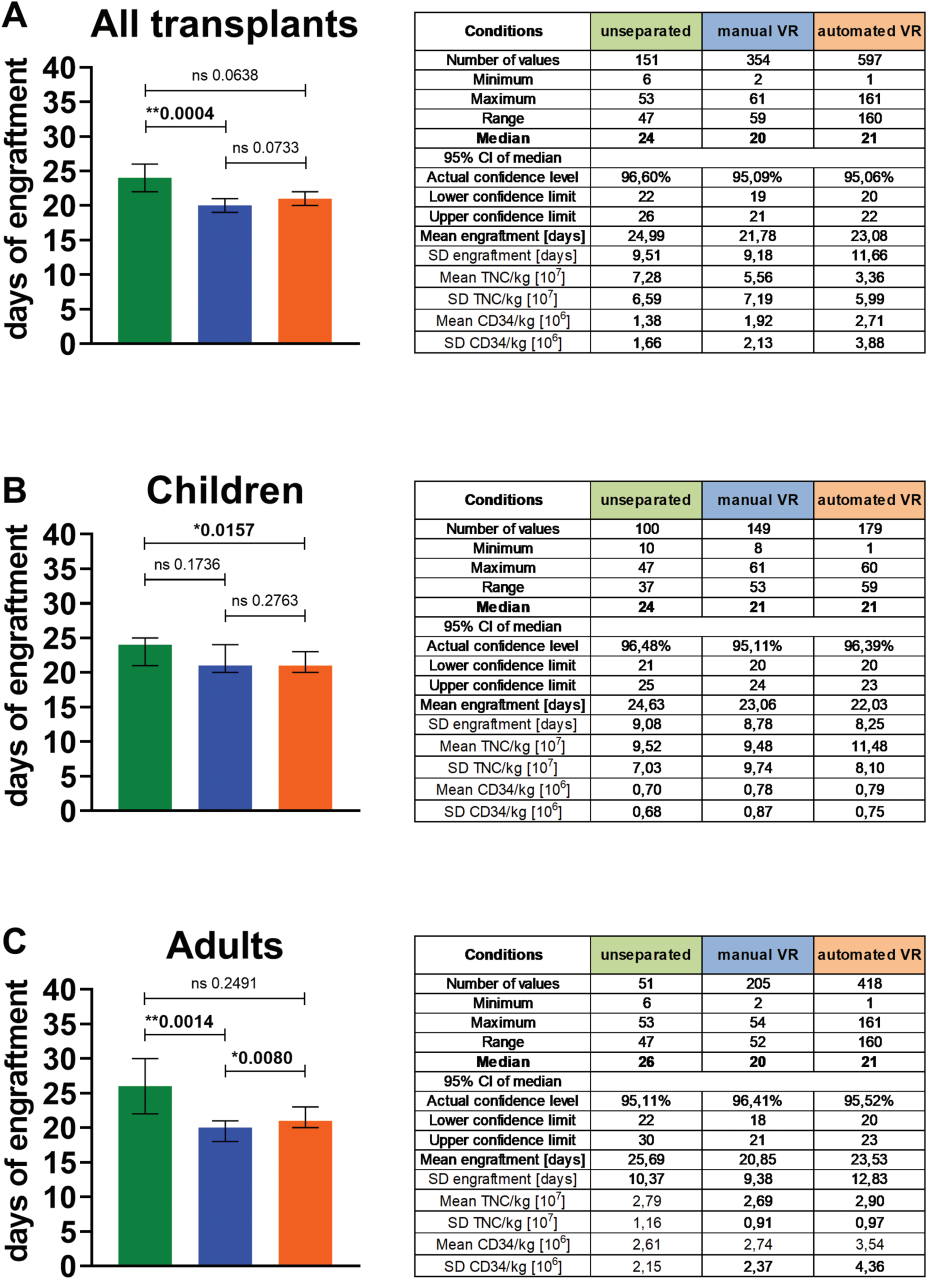
Time to neutrophil engraftment related to patient age days until engraftment 5E8 are presented as median with 95% confidence interval for: (**A**) all released transplants, (**B**) transplanted children, and (**C**) transplanted adults. Engraftment data were categorized by distinct processing methods applied for unseparated CBUs processed from 1993 to 1997 (green), manual volume-reduced (manual VR) CBUs processed from 1997 to 2005 (blue) and automated volume-reduced (automated VR) CBUs processed from 2005 to date (orange). An unpaired 2-tailed *t*-test was performed to determine significant differences. According statistics and related dose of TNC/kg and CD34/kg are given in the right panel.

The 2 volume-reduced groups reveal early engraftment for neutrophils 5E8 with 20 days for manual VR and 21 days for automated VR versus 24 days for unseparated units analyzing all transplants (Fig. 7A). A significant difference was detected in between the unseparated versus manual VR group with a ***P-value of .0004. Similar results were gained by comparison of transplanted children (Fig. 7B) with 21 days for manual VR and automated VR versus 24 days for unseparated units. A significant difference was determined in the unseparated versus the automated VR group with a *P-value of .0157. In transplanted adults (Fig. 7C), the latest engraftment was determined for unseparated units with a median of 26 versus 20 days of engraftment for manual VR and 21 days for automated VR. Significance was observed by comparing unseparated versus manual VR group (**.0014) and manual VR versus automated VR group (**.0080). These results clearly show an earlier engraftment for volume-reduced CBUs as compared to unseparated CBUs. Respective TNC counts/kg and CD34 counts/kg show a decrease of engraftment time with higher cell counts applied for transplantation.

In Supplementary Fig. S2A-S2F, the detailed results of days until engraftment (presented here as categories) versus TNC count/kg and versus CD34^+^ count/kg confirm the prior result.

Then, it was analyzed if the engraftment time, TNC count/kg and CD34^+^ count/kg are potentially affected by longer duration in cryopreserved storage as a measure of stability. Therefore, the outcomes are split by a 2-year period of storage related to the distinct processing methods (Supplementary Fig. S3A-S3C). No significant effect was determined for longer storage times. However, sample numbers were low for the longest stored and released transplants (Supplementary Tables 4A-C).

Finally, the transplant outcomes were analyzed by performing the cumulative incidence of neutrophil engraftment 42 days and 60 days after transplantation for all 3 processing methods (Supplementary Fig. S4). Although not significant, this confirmed previous results in line with the later engraftment for unseparated CBUs as compared to both volume-reduced groups.

## Discussion

This work clearly demonstrates the high quality of CBUs stored at the CBB over 29 years of cryopreservation. A detailed analysis was conducted according to distinct processing methods. It can be clearly stated that the oldest unseparated units still show a very high viability (88.91 ± 5.01% for TNC viability, 90.73 ± 3.69 for CD34^+^7AAD^−^ viability, and 69.09 ± 5.38% for CD45^+^7AAD^−^ viability) after 29 years of cryopreservation. For both volume-reduced groups similar means are determined after 25 (manual) and 18 years (automated) of cryopreservation. Despite this high viability of the oldest CBUs, preferably transplants cryopreserved within the last 5-7 years are requested by transplant centers. This is confirmed by the literature stating that the duration of storage is used as one of the preferred selection criteria.^21^ This might be in particular due to improved high-resolution HLA-sequencing for HLA-A, -B, -C, -DR, DQ, DP (Next Generation Sequencing [NGS] included at the CBB Düsseldorf since 2016) on the segment, the initial CBU aliquot and maternal blood in parallel saving time by providing the results at release for storage confirming the haplotype. Moreover, extensive virus testing is performed, and extended anamnestic data are requested in the maternal risk questionnaire. All these factors lead to the preferred request of younger CBUs but is not causative to the quality of the CBU itself. The results presented here confirm the preferred request of younger CBUs with a high TNC count range (≥15 × 10^8^) for automated volume-reduction in line with the storage time and criteria for CBU selection followed by the transplant centers or the registries.^22^ In addition to the described preferences at request one predictive parameter for better engraftment is the infused viable CD34^+^ cell dose of the dominant unit in double cord blood transplantation (CBT).^23^ For single units after CBT, a correlation is demonstrated between the prefreeze TNC count and engraftment,^4,24^ whereas for double-unit CBT the post-thaw CD34^+^ viability is associated with better engraftment.^25,26^ In other approaches, the dose of infused CFUs is a better predictive factor for transplant outcomes over TNC or CD34^+^ count,^27,28^ whereas Castillo et al did not find an association. The only marginal significant predictor (*P-value .04480) within our datasets was the CD34^+^ count after processing resulting in a mean of 7.91 × 10^6^ for non-engrafted CBUs (*n* = 88) as compared to a mean of 9.41 × 10^6^ for engrafted CBUs (*n* = 835) (Supplementary Table S5). They demonstrated the clonogenic efficiency together with the post-thaw viable CD45^+^ count as predictor for engraftment.^29^ However, possible inter-laboratory variations of techniques must be discussed.^30,31^ Therefore, even banking practices are recommended to be incorporated into unit selection.^23^

The described preferences exclude older highly qualified CBUs from transplantation applications. Nevertheless, older CBUs can be easily requalified for the establishment of ATMPs, since retained samples are available, and the donors are adults at time of necessary re-consenting. The CBB Düsseldorf was one of the first banks developing a feasible strategy for establishment of an IPSC master stem cell bank generated from banked HLA-homozygous CBUs in 2020 which was funded by the BMBF under the acronym HLA-iPS-GMP subsequently resulting in the recent Heal project (**H**LA-homozygous iPSC-cardiomyocyt**E A**ggregate manufacturing techno**L**ogies for allogenic cell therapy to the heart; Grant agreement ID: 101056712).^32^ Similar approaches are described by others and provide important alternatives for unreleased transplants.^33-38^

Unseparated CBUs with a high volume for transplantation in children must be washed due to the high number of erythrocytes and a high DMSO content. A possible impact on CBU quality is often dependent on the individual processing methods as described eg, for the volume-reduction with hydroxyethyl starch or non-hydroxyethyl starch.^39^ However, the stability data presented here confirms the high quality of older CBUs despite longer storage time in relation to distinct processing methods applied at the CBB Düsseldorf.

Unseparated CBUs and manual volume-reduced CBUs revealed lower mean TNC count ranges as compared to automated volume-reduced CBUs. As of 2013, the TNC count limit was increased up 16 × 10^8^ likely impacting the higher mean TNC count.

Regarding the transplant outcomes, a retrospective analysis from follow-up data of released transplants re-confirmed that a higher TNC count/kg is associated with an earlier engraftment in children. This effect is correlating to the fact that children are typically transplanted with a higher TNC count of ≥ 3 × 10^7^ TNC/kg as compared to ≥ 2 × 10^7^ TNC/kg in adults. Older CBUs had lower TNC counts leading to a preferred transplantation of children in earlier days, in accordance to the requirements for the TNC/kg. This is in line with the recent literature demonstrating the importance of applied cell dose in transplanted children.^7^ However, in adults, this correlation was not confirmed, maybe due to the lower cell dose (≥2 × 10^7^ TNC/kg in adults versus ≥ 3 × 10^7^ TNC/kg applied in children). In summary, the CBB Düsseldorf stores high quality CBUs, which are released for transplantation confirmed by the internal stability program. The stability of unseparated CBUs is guaranteed for 29 years and volume-reduced CBUs can be cryopreserved in liquid nitrogen for 25 years. Follow-up data from already released transplants demonstrate earlier engraftment in both volume-reduced groups versus unseparated CBUs processed from 1993-1997 correlating to higher TNC and CD34^+^ counts/kg applied for transplantation.

## Conclusion

This study clearly demonstrates that the time of cryopreservation has no significant detrimental effect on engraftment after transplantation and confirms the high quality of CBUs demonstrated by stability data and post-thaw analyses deriving from released transplants.

## Supplementary Material

szad071_suppl_Supplementary_Tables_1-5_Figures_1-4

## Data Availability

The data that support the findings of this study are available from the corresponding author upon reasonable request.
