# Supplementary material for: Long-Term Stability of Cord Blood Units After 29 Years of Cryopreservation: Follow-Up Data From the José Carreras Cord Blood Bank
Source: Stem Cells Transl Med. 2023 Nov 4;13(1):30–42. doi: 10.1093/stcltm/szad071 (PMC10785215; doi:10.1093/stcltm/szad071)
Supplement: szad071_suppl_Supplementary_Tables_1-5_Figures_1-4 [file szad071_suppl_supplementary_tables_1-5_figures_1-4.pdf]

Supplementary Table 1. Specifications according to FACT and PEI

Parameter	FACT standards 7th edition		PEI/Internal CBB Limits	
	**Post-processing prior to cryopreservation**	**After thaw**	**Post-processing prior to cryopreservation**	**After thaw**
TNC count [10^8]	**≥5 x 10^8**	not specified	**≥5 x 10^8**	**≥5 x 10^8**
TNC Recovery [%]	**≥85%**	not specified	**≥60%**	not specified
TNC Viability [%]	**≥60%**	not specified	**≥85%**	**≥60%**
Viable $CD34^+$ count [10^6]	**≥1.25 x 10^6**	not specified	**≥1.25 x 10^6**	not specified
Viability of $CD34^+$ cells	not specified	**≥70%**	**≥85%**	**≥70%**
Viability of $CD45^+$ cells	not specified	not specified	not specified	**≥40%**
CFU assay	not specified	**Growth**	**≥1 colony**	**≥1 colony**

Remark: Some FACT specifications changed within the distinct editions over time. As an example: The viability of the $CD45^+7AAD^-$ population has an internal limit here at the ITZ-UKD of **≥40%** after thaw. This limit was adopted from prior FACT specification 6th edition, since it was removed in the 7th edition.

Supplementary Table 2. Raw data tables according to expiration time

A) Extension of expiration time after 338 months (n=11) for unseparated CBUs

CBU sample	Initial CB Volume (incl. CPD) [ml]	Transport duration [hours]	Minimum transport temperature [°C]	Maximum transport temperature [°C]	Cryopreservation date [dd.mm.yyyy]	Volume of transplant (incl. freezing solution) [ml]	Number of bags [n]	Storage time [months]	TNC count (incl. erythroblasts) after thaw $[10^8]$	TNC recovery (incl. erythroblasts) after thaw [%]	TNC viability (incl. erythroblasts) after thaw [%]	Viability CD34$^+$7AAD$^-$ after thaw [%]	Viability CD45$^+$7AAD$^-$ after thaw [%]	CD34$^+$ count (related to TNC count incl. erythroblasts) after thaw $[10^6]$	CFC count after thaw $[10^6]$
1	90	0,1	18	26	24.09.1993	180	2	350	18,41	130,32	80	90	69	9,87	5,57
2	106	0,1	18	26	17.01.1994	212	3	346	16,98	104,71	98	85	74	2,28	4,69
3	75	0,1	18	26	15.08.1994	150	2	341	12,02	90,00	90	87	73	1,98	2,16
4	95	0,1	18	26	16.09.1994	190	2	340	14,82	113,87	95	94	66	28,90	7,45
5	108	0,1	18	26	12.10.1994	216	3	339	15,49	112,03	90	93	67	8,64	7,49
6	127	0,1	18	26	11.12.1994	254	3	337	19,05	102,04	90	91	66	4,00	8,40
7	141	0,1	18	26	07.02.1995	282	3	335	18,02	96,82	85	89	72	4,94	8,12
8	106	0,1	18	26	14.02.1995	212	2	335	18,51	131,28	90	92	79	6,83	11,76
9	89	0,1	18	26	30.03.1995	178	2	334	12,39	104,66	90	95	71	3,41	6,22
10	98	0,1	18	26	18.09.1995	196	2	328	19,23	94,78	85	86	62	5,31	8,15
11	105	0,1	18	26	25.09.1995	210	2	328	14,62	94,69	85	96	61	2,95	8,12

B) Extension of expiration time after 91 months (n=10) for manual volume-reduced CBUs

CBU sample	Initial CB Volume (incl. CPD) [ml]	Transport duration [hours]	Cryopreservation date [dd.mm.yyyy]	Volume of transplant (incl. freezing solution) [ml]	Number of bags [n]	Storage time [months]	TNC count (incl. erythroblasts) after thaw $[10^8]$	TNC recovery (incl. erythroblasts) after thaw [%]	TNC viability (incl. erythroblasts) after thaw [%]	Viability CD34$^+$7AAD$^-$ after thaw [%]	Viability CD45$^+$7AAD$^-$ after thaw [%]	CD34$^+$ count (related to TNC count incl. erythroblasts) after thaw $[10^6]$	CFC count after thaw $[10^6]$
1	77	0,3	08.12.1997	25,8	1	92	5,44	102,68	97	n.d.	n.d.	0,80	0,71
2	83	0,3	22.12.1997	25,8	1	92	7,17	91,52	97	n.d.	n.d.	1,65	0,92
3	105	0,3	23.12.1997	29,8	1	92	6,35	94,98	95	n.d.	n.d.	0,94	0,73
4	82	0,5	06.01.1997	25,8	1	91	6,84	96,80	97	n.d.	n.d.	0,68	0,87
5	76	1,5	06.01.1998	25,8	1	91	4,31	92,78	97	n.d.	n.d.	0,65	0,68
6	94	1,5	08.01.1998	29,8	1	91	7,60	111,84	97	n.d.	n.d.	0,24	0,75
7	79	1	09.01.1998	25,8	1	91	4,70	92,27	97	n.d.	n.d.	1,55	0,53
8	105	1,15	10.01.1998	53,2	2	91	4,45	92,01	95	n.d.	n.d.	2,76	1,24
9	101	0,3	15.01.1998	25,8	1	91	6,86	99,07	97	n.d.	n.d.	0,90	0,84
10	83	0,5	19.01.1997	25,8	1	91	6,24	106,14	97	n.d.	n.d.	0,38	0,62

C) Extension of expiration time after 142 months (n=10) for manual volume-reduced CBUs

CBU sample	Initial CB Volume (incl. CPD) [ml]	Transport duration [hours]	Cryopreservation date [dd.mm.yyyy]	Volume of transplant (incl. freezing solution) [ml]	Number of bags [n]	Storage time [months]	TNC count (incl. erythroblasts) after thaw $[10^8]$	TNC recovery (incl. erythroblasts) after thaw [%]	TNC viability (incl. erythroblasts) after thaw [%]	Viability CD34$^+$7AAD$^-$ after thaw [%]	Viability CD45$^+$7AAD$^-$ after thaw [%]	CD34$^+$ count (related to TNC count incl. erythroblasts) after thaw $[10^6]$	CFC count after thaw $[10^6]$
11	98	1h	14.04.1998	29,8	1	142	7,08	88,51	90	n.d.	n.d.	2,90	2,08
12	102	1h	19.04.1998	29,8	1	142	11,40	83,63	90	n.d.	n.d.	6,16	3,40
13	163	0,5	21.04.1998	53,2	2	142	12,80	87,55	85	n.d.	n.d.	4,35	0,10
14	127	1h	24.04.1998	25,8	1	142	10,30	88,53	85	n.d.	n.d.	2,68	1,60
15	117	1h	27.04.1998	29,8	1	142	9,84	65,39	98	n.d.	n.d.	1,48	2,76
16	98	1h	27.04.1998	53,2	2	142	11,02	77,17	90	n.d.	n.d.	8,15	3,59
17	98	0,5h	27.04.1998	53,2	2	142	6,41	76,69	85	n.d.	n.d.	1,60	1,91
18	81	1,5h	28.04.1998	25,8	1	142	9,36	73,07	98	n.d.	n.d.	1,03	1,52
19	105	1,5h	28.04.1998	25,8	1	142	10,80	83,08	80	n.d.	n.d.	2,92	1,52
20	90	1,5h	28.04.1998	25,8	1	142	6,03	67,72	98	n.d.	n.d.	0,78	0,65

D) Extension of expiration time after 187 months (n=14) for manual volume-reduced CBUs

CBU sample	Initial CB Volume (incl. CPD) [ml]	Transport duration [hours]	Cryopreservation date [dd.mm.yyyy]	Volume of transplant (incl. freezing solution) [ml]	Number of bags [n]	Storage time [months]	TNC count (incl. erythroblasts) after thaw $[10^8]$	TNC recovery (incl. erythroblasts) after thaw [%]	TNC viability (incl. erythroblasts) after thaw [%]	Viability CD34$^+$7AAD$^-$ after thaw [%]	Viability CD45$^+$7AAD$^-$ after thaw [%]	CD34$^+$ count (related to TNC count incl. erythroblasts) after thaw $[10^6]$	CFC count after thaw $[10^6]$
21	85	1h	05.12.1997	29,8	1	187	7,57	95,32	90	n.d.	n.d.	2,04	0,71
22	93	1h	05.12.1997	29,8	1	187	8,20	86,92	95	n.d.	n.d.	3,12	1,42
23	105	0,5	12.12.1997	29,8	1	187	10,82	97,77	90	n.d.	n.d.	6,49	1,13
24	122	1h	22.12.1997	25,8	1	187	18,03	97,92	85	n.d.	n.d.	16,77	1,26
25	111	0,5	15.01.1998	29,8	1	186	9,12	89,19	97	n.d.	n.d.	2,19	1,17
26	90	1,5h	16.01.1998	25,8	1	186	7,82	91,92	98	n.d.	n.d.	2,42	1,18
27	79	1h	19.01.1998	25,8	1	186	8,28	106,88	92	n.d.	n.d.	2,07	0,71
28	101	1,5h	21.01.1997	25,8	1	186	10,29	95,37	95	n.d.	n.d.	2,57	1,13
29	114	1,5h	25.01.1998	29,8	1	186	7,13	89,00	98	n.d.	n.d.	3,57	1,44
30	126	1h	25.01.1998	29,8	1	186	10,10	87,51	98	n.d.	n.d.	3,94	1,53
31	87	1,5h	29.01.1998	53,2	2	186	10,58	94,71	96	n.d.	n.d.	3,28	3,31
32	97	0,5h	29.01.1198	53,2	2	186	14,79	78,81	95	n.d.	n.d.	4,14	4,95
33	106	0,1h	02.02.1998	25,8	1	185	8,75	79,94	98	n.d.	n.d.	2,45	3,53
34	81	1h	02.02.1998	25,8	1	185	8,67	78,63	96	n.d.	n.d.	2,51	2,25

E) Extension of expiration time after 210 months (n=10) for manual volume-reduced CBUs

**CBU sample**	**Initial CB Volume** (incl. CPD) **[ml]**	**Transport duration [hours]**	**Cryopreservation date [dd.mm.yyyy]**	**Volume of transplant** (incl. freezing solution) **[ml]**	**Number of bags [n]**	**Storage time [months]**	**TNC count** (incl. erythroblasts) **after thaw [10^8]**	**TNC recovery** (incl. erythroblasts) **after thaw [%]**	**TNC viability** (incl. erythroblasts) **after thaw [%]**	**Viability $CD34^+7AAD^-$ after thaw [%]**	**Viability $CD45^+7AAD^-$ after thaw [%]**	**$CD34^+$ count** (related to TNC count incl. erythroblasts) **after thaw [10^6]**	**CFC count after thaw [10^6]**
35	102	0	26.06.1998	25,8	1	210	9,05	88,47	80	n.d.	n.d.	6,43	1,19
36	98	0,5	14.07.1998	29,8	1	209	11,07	91,71	90	n.d.	n.d.	4,02	1,60
37	97	1	20.07.1998	25,8	1	209	10,40	87,39	80	n.d.	n.d.	1,79	0,48
38	197	1	20.08.1998	53,2	2	208	24,92	108,30	90	n.d.	n.d.	12,46	2,62
39	111	1	03.09.1998	29,8	1	207	10,44	87,73	90	n.d.	n.d.	3,56	1,38
40	117	0,5	23.10.1998	29,8	1	206	11,16	91,55	90	n.d.	n.d.	5,12	1,21
41	95	10	06.11.1998	29,8	1	205	10,62	96,20	90	n.d.	n.d.	1,93	1,19
42	118	1h	07.04.1999	25,8	1	200	9,91	82,51	90	n.d.	n.d.	2,35	0,98
43	96	0,5h	27.09.1999	25,8	1	195	11,52	78,37	90	n.d.	n.d.	3,24	1,36
44	78	1h	13.10.1999	25,8	1	194	11,95	83,57	90	n.d.	n.d.	6,35	1,53

F) Extension of expiration time after 232 months (n=10) for manual volume-reduced CBUs

**CBU sample**	**Initial CB Volume** (incl. CPD) **[ml]**	**Transport duration [hours]**	**Cryopreservation date [dd.mm.yyyy]**	**Volume of transplant** (incl. freezing solution) **[ml]**	**Number of bags [n]**	**Storage time [months]**	**TNC count** (incl. erythroblasts) **after thaw [10^8]**	**TNC recovery** (incl. erythroblasts) **after thaw [%]**	**TNC viability** (incl. erythroblasts) **after thaw [%]**	**Viability $CD34^+7AAD^-$ after thaw [%]**	**Viability $CD45^+7AAD^-$ after thaw [%]**	**$CD34^+$ count** (related to TNC count incl. erythroblasts) **after thaw [10^6]**	**CFC count after thaw [10^6]**
45	80	0,1	23.12.1997	25,8	1	232	10,75	102,79	85	97	86	2,90	1,67
46	149	0,1	03.01.1998	25,8	1	231	8,97	84,08	80	93	83	3,77	2,16
47	92	1	09.01.1998	29,8	1	231	8,76	87,32	75	91	57	2,54	0,72
48	101	1	10.01.1998	29,8	1	231	8,72	87,13	90	91	55	2,44	1,12
49	120	0,5	14.01.1998	25,8	1	231	15,60	96,47	80	97	59	4,68	2,19
50	125	1	14.01.1998	53,2	2	231	10,61	91,53	80	92	41	3,40	0,75
51	124	1	16.01.1998	53,2	2	231	12,19	94,85	80	95	73	4,75	1,07
52	102	1	19.01.1998	25,8	1	231	10,84	94,54	90	88	65	3,25	1,71
53	115	0,1	24.01.1998	25,8	1	231	12,60	102,56	85	98	85	3,78	2,87
54	86	1	28.01.1998	29,8	1	231	8,37	82,25	75	82	65	1,00	0,58

G) Extension of expiration time after 252 months (n=10) for manual volume-reduced CBUs

**CBU sample**	**Initial CB Volume** (incl. CPD) **[ml]**	**Transport duration [hours]**	**Cryopreservation date [dd.mm.yyyy]**	**Volume of transplant** (incl. freezing solution) **[ml]**	**Number of bags [n]**	**Storage time [months]**	**TNC count** (incl. erythroblasts) **after thaw [10^8]**	**TNC recovery** (incl. erythroblasts) **after thaw [%]**	**TNC viability** (incl. erythroblasts) **after thaw [%]**	**Viability $CD34^+7AAD^-$ after thaw [%]**	**Viability $CD45^+7AAD^-$ after thaw [%]**	**$CD34^+$ count** (related to TNC count incl. erythroblasts) **after thaw [10^6]**	**CFC count after thaw [10^6]**
55	136	1	19.12.1997	29,8	1	252	12,78	92,61	85	93	70	4,35	1,71
56	77	2	05.01.1998	25,8	1	252	8,40	116,34	90	93	73	1,34	1,32
57	83	1	09.01.1998	25,8	1	251	8,65	95,79	80	78	54	2,42	0,55
58	96	0,5	14.01.1998	25,8	1	251	7,51	92,37	80	72	42	1,65	0,18
59	97	1	14.01.1998	53,2	2	252	11,12	114,00	89	90	71	1,50	2,51
60	114	1	31.01.1998	29,8	1	251	13,95	99,01	80	86	50	7,25	0,46
61	143	0,5	03.02.1998	53,2	2	250	15,89	123,24	80	85	60	7,31	2,51
62	115	1	04.02.1998	25,8	1	251	11,46	98,33	90	84	67	3,21	1,44
63	102	1	05.02.1998	53,2	2	251	12,36	109,81	88	92	80	5,56	2,76
64	129	1	06.02.1998	53,2	2	251	11,58	96,74	90	94	82	3,35	3,80

H) Extension of expiration time after 265 months (n=8) for manual volume-reduced CBUs

**CBU sample**	**Initial CB Volume** (incl. CPD) **[ml]**	**Transport duration [hours]**	**Cryopreservation date [dd.mm.yyyy]**	**Volume of transplant** (incl. freezing solution) **[ml]**	**Number of bags [n]**	**Storage time [months]**	**TNC count** (incl. erythroblasts) **after thaw [10^8]**	**TNC recovery** (incl. erythroblasts) **after thaw [%]**	**TNC viability** (incl. erythroblasts) **after thaw [%]**	**Viability $CD34^+7AAD^-$ after thaw [%]**	**Viability $CD45^+7AAD^-$ after thaw [%]**	**$CD34^+$ count** (related to TNC count incl. erythroblasts) **after thaw [10^6]**	**CFC count after thaw [10^6]**
65	121	1	06.02.1998	25,8	1	265	10,22	74,73	80	92	86	2,04	0,90
66	94	1	06.02.1998	29,8	1	265	9,90	94,03	85	95	74	1,58	1,68
67	81	1	06.02.1998	53,2	2	265	8,55	92,63	80	94	75	0,94	1,61
68	78	0,5	08.02.1998	29,8	1	265	12,06	101,06	80	96	85	5,79	1,74
69	77	1	09.02.1998	25,8	1	265	8,27	83,89	80	92	81	0,91	0,80
70	123	0,5	09.02.1998	29,8	1	265	12,87	78,09	80	96	74	2,45	1,81
71	96	0,5	13.02.1998	25,8	1	265	12,79	79,26	80	94	82	6,65	1,41
72	88	1	13.02.1998	25,8	1	265	10,69	97,15	90	93	86	3,21	1,59

I) Extension of expiration time after 281 months (n=12) for manual volume-reduced CBUs

CBU sample	Initial CB Volume (incl. CPD) [ml]	Transport duration [hours]	Cryopreservation date [dd.mm.yyyy]	Volume of transplant (incl. freezing solution) [ml]	Number of bags [n]	Storage time [months]	TNC count (incl. erythroblasts) after thaw [10^8]	TNC recovery (incl. erythroblasts) after thaw [%]	TNC viability (incl. erythroblasts) after thaw [%]	Viability $CD34^+7AAD^-$ after thaw [%]	Viability $CD45^+7AAD^-$ after thaw [%]	$CD34^+$ count (related to TNC count incl. erythroblasts) after thaw [10^6]	CFC count after thaw [10^6]
73	95	0,5	17.02.1998	29,8	1	281	12,87	84,18	80	94	72	6,95	1,44
74	122	0,1	17.02.1998	25,8	1	281	10,37	89,49	90	96	83	1,56	0,76
75	78	0,5	17.02.1998	25,8	1	281	5,98	80,93	90	94	81	1,44	0,36
76	119	1	17.02.1998	29,8	1	281	9,81	80,94	70	94	68	4,02	1,54
77	83	0,5	17.02.1998	29,8	1	281	8,08	91,76	60	95	74	1,45	0,72
78	99	1	17.02.1998	29,8	1	281	7,75	91,47	70	91	69	0,70	0,46
79	94	0,5	05.03.1998	53,2	2	280	8,12	92,46	70	87	66	0,52	0,29
80	149	0,5	07.03.1998	53,2	2	280	12,49	92,36	80	95	78	3,60	1,09
81	91	1	06.03.1998	29,8	1	280	9,18	93,52	50	90	59	1,63	1,04
82	87	1	11.03.1998	29,8	1	280	9,54	85,12	70	94	74	3,58	1,41
83	114	1	16.03.1998	53,2	2	280	13,24	101,00	70	86	62	2,54	0,60
84	103	0,1	20.03.1998	53,2	2	280	9,72	91,84	70	97	64	1,91	1,32

J) Extension of expiration time after 286 months (n=7) for manual volume-reduced CBUs

CBU sample	Initial CB Volume (incl. CPD) [ml]	Transport duration [hours]	Cryopreservation date [dd.mm.yyyy]	Volume of transplant (incl. freezing solution) [ml]	Number of bags [n]	Storage time [months]	TNC count (incl. erythroblasts) after thaw [10^8]	TNC recovery (incl. erythroblasts) after thaw [%]	TNC viability (incl. erythroblasts) after thaw [%]	Viability $CD34^+7AAD^-$ after thaw [%]	Viability $CD45^+7AAD^-$ after thaw [%]	$CD34^+$ count (related to TNC count incl. erythroblasts) after thaw [10^6]	CFC count after thaw [10^6]
85	76	0,5	03.02.1998	25,8	1	287	9,13	89,05	60	95	61	2,56	1,93
86	93	0,5	07.03.1998	25,8	1	286	9,44	80,40	60	90	55	1,29	0,67
87	99	0,1	11.03.1998	29,8	1	286	10,74	89,50	70	86	58	5,92	2,27
88	111	1	13.03.1998	29,8	1	286	11,16	86,11	50	94	50	3,34	1,44
89	86	1	17.03.1998	25,8	1	286	9,36	88,09	80	94	80	2,99	1,47
90	115	0,5	26.03.1998	29,8	1	286	14,85	96,68	70	95	75	5,32	1,68
91	92	1	26.03.1998	53,2	2	286	9,98	105,60	90	94	83	2,17	1,87

K) Extension of expiration time after 300 months (n=9) for manual volume-reduced CBUs

CBU sample	Initial CB Volume (incl. CPD) [ml]	Transport duration [hours]	Cryopreservation date [dd.mm.yyyy]	Volume of transplant (incl. freezing solution) [ml]	Number of bags [n]	Storage time [months]	TNC count (incl. erythroblasts) after thaw [10^8]	TNC recovery (incl. erythroblasts) after thaw [%]	TNC viability (incl. erythroblasts) after thaw [%]	Viability $CD34^+7AAD^-$ after thaw [%]	Viability $CD45^+7AAD^-$ after thaw [%]	$CD34^+$ count (related to TNC count incl. erythroblasts) after thaw [10^6]	CFC count after thaw [10^6]
92	123	0,5	13.12.1997	25,8	1	300	11,31	97,22	90	91	69	1,47	0,94
93	105	0,5	18.12.1997	25,8	1	300	13,26	96,11	90	92	76	2,52	1,47
94	84	0,1	18.12.1997	25,8	1	300	7,64	94,94	90	78	78	1,45	0,89
95	108	1	14.01.1998	53,2	2	300	8,18	104,16	80	94	75	2,95	2,19
96	99	1	26.01.1998	25,8	1	300	7,96	98,40	88	84	75	0,56	1,55
97	106	0,5	28.01.1998	53,2	2	300	8,59	92,08	60	85	64	1,03	0,26
98	81	1	29.01.1998	29,8	1	300	8,96	96,02	90	89	83	2,96	1,42
99	121	1	09.02.1998	29,8	1	300	11,52	102,13	80	87	59	4,03	1,62
100	88	1	13.02.1998	53,2	2	300	6,95	99,68	90	93	84	1,11	1,96

L) Extension of expiration time after 211 months (n=11) for automated volume-reduced CBUs

CBU sample	Initial CB Volume (incl. CPD) [ml]	Transport duration [hours]	Cryopreservation date [dd.mm.yyyy]	Volume of transplant incl. aliquots [ml]	Number of bags [n]	Storage time [months]	TNC count (incl. erythroblasts) after thaw [10^8]	TNC recovery (incl. erythroblasts) after thaw [%]	TNC viability (incl. erythroblasts) after thaw [%]	Viability $CD34^+7AAD^-$ after thaw [%]	Viability $CD45^+7AAD^-$ after thaw [%]	$CD34^+$ count (related to TNC count incl. erythroblasts) after thaw [10^6]	CFC count after thaw [10^6]
1	101,2	1	06.10.2005	29,8	1	212	15,73	93,39	80	85	64	6,29	2,35
2	100,2	1	06.10.2005	29,8	1	212	13,50	91,91	82	81	86	3,78	2,00
3	130,2	1,5	07.10.2005	53,2	2	212	11,11	92,48	92	93	80	8,22	4,23
4	110,2	1	08.10.2005	25,8	1	212	11,38	89,41	90	91	78	2,96	1,16
5	102,2	1	10.10.2005	29,8	1	212	12,25	84,49	90	88	83	4,90	3,04
6	107,2	0,5	10.10.2005	29,8	1	212	14,30	93,42	90	91	86	2,43	2,15
7	105,2	1	13.10.2005	29,8	1	212	14,57	92,56	90	94	86	2,62	2,34
8	107,2	1	13.10.2005	29,8	1	212	16,54	90,91	90	96	68	2,48	2,34
9	105,2	1	21.10.2005	29,8	1	212	19,85	99,87	89	90	77	4,17	2,65
10	127,1	1	23.10.2005	25,8	1	212	13,55	88,84	92	90	86	8,13	2,49
11	126,2	1,5	16.05.2006	53,2	2	205	15,51	81,36	90	91	79	2,17	4,14

Supplementary Table 3: Post-thaw analysis of relevant parameters in released transplants categorized after the distinct processing methods

Parameter	Processing method	Number of values	Minimum	Maximum	Range	Mean	Std. Deviation
TNC Recovery after thaw [%]	unseparated	188	46.96	124.90	77.91	91.59	12.37
	manual volume-reduced	484	52.58	148.50	95.87	84.32	9.29
	automated volume-reduced	756	46.29	219.70	173.50	88.87	10.47
TNC count after thaw [10^8]	unseparated	188	2.72	38.40	35.68	14.15	6.74
	manual volume-reduced	484	0.83	36.30	35.47	14.08	5.09
	automated volume-reduced	756	1.27	55.40	54.13	17.83	6.41
$CD34^+$ count after thaw [10^6]	unseparated	187	0.06	33.20	33.14	5.80	5.68
	manual volume-reduced	481	0.40	41.90	41.50	5.91	5.20
	automated volume-reduced	759	0.26	312.00	311.70	9.82	17.39
TNC Viability after thaw [%]	unseparated	178	70	99	29	92.99	4.77
	manual volume-reduced	81	40	99	59	88.83	9.94
	automated volume-reduced	571	60	99	39	89.99	7.38
$CD34^+7AAD^-$ Viability after thaw [%]	unseparated		no specification for CD34+/CD45+ viability determination at that time				
	manual volume-reduced	17	84	100	16	94.06	4.88
	automated volume-reduced	257	73	100	27	92.80	5.58
$CD45^+7AAD^-$ Viability after thaw [%]	unseparated		no specification for CD34+/CD45+ viability determination at that time				
	manual volume-reduced	17	64	90	26	78.53	7.02
	automated volume-reduced	257	45	88	43	68.97	9.69
CFC count after thaw [10^6]	unseparated	201	0.28	17.63	17.35	2.83	2.71
	manual volume-reduced	461	0.10	13.86	13.76	1.51	1.56
	automated volume-reduced	464	0.06	11.35	11.29	2.80	1.53

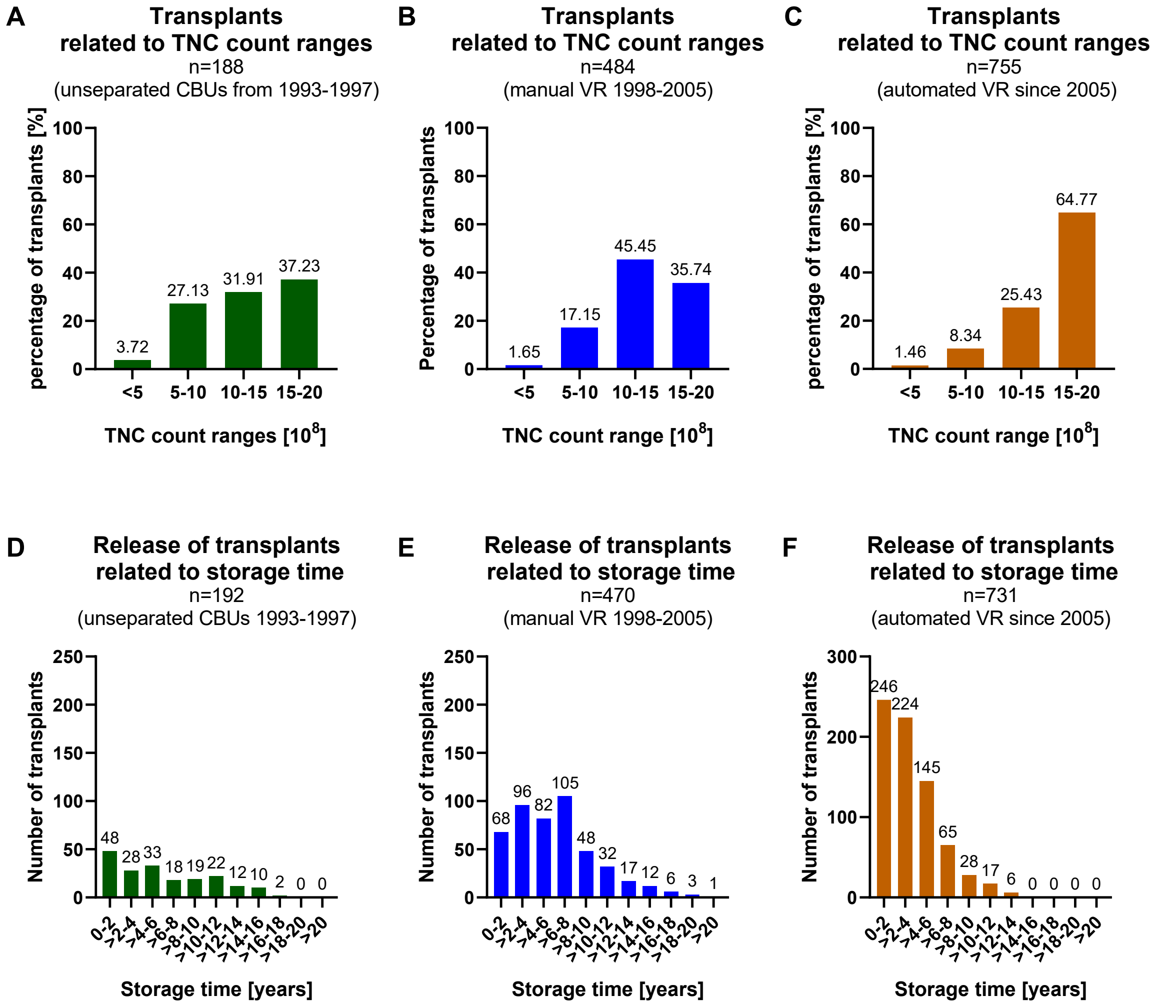

Supplementary Figure 1: TNC count ranges and preferred release of transplants according to storage time and distinct processing methods

Frequencies of distribution of TNC count ranges are presented for (A) unseparated CBUs processed from 1993-1997, (B) CBUs processed from 1998-2005 by manual volume-reduction and (C) CBUs processed since 2005 by automated volume-reduction. The storage time is presented as the difference between the CB birth date and the TX date for (D) unseparated CBUs processed from 1993-1997, (E) manual volume-reduced CBUs processed from 1998-2005 and (F) automated volume-reduced CBUs processed since 2005.

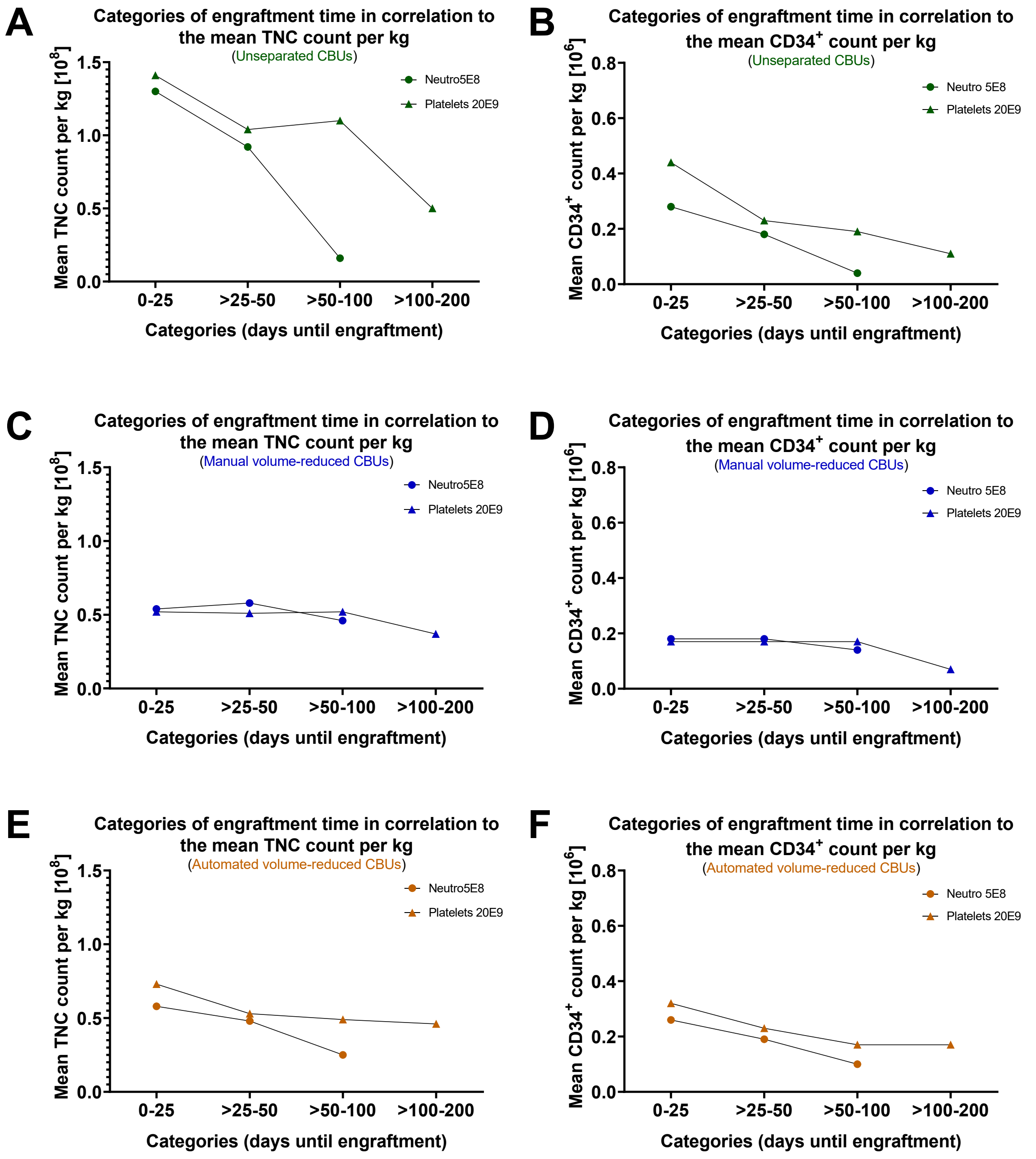

Supplementary Figure 2: Neutrophil and platelets engraftment versus TNC count and CD34⁺ count per weight

The date of engraftment is considered as the first of 3 consecutive days of neutrophils ≥ 5x10e8/L, without evidence of autologous reconstitution and the date of platelets recovery is considered as the first day platelets ≥20x10e9/L without platelets transfusion during 7 consecutive days and without evidence of autologous reconstitution or graft rejection in the first 100 days. Frequencies of distribution are performed for the days until engraftment versus the respective mean TNC count/kg (A, C, E) and CD34⁺ count/kg (B, D, F). Results are given separately for three distinct processing methods: unseparated CBUs (green), manual volume-reduced CBUs (blue) and automated volume-reduced CBUs (orange).

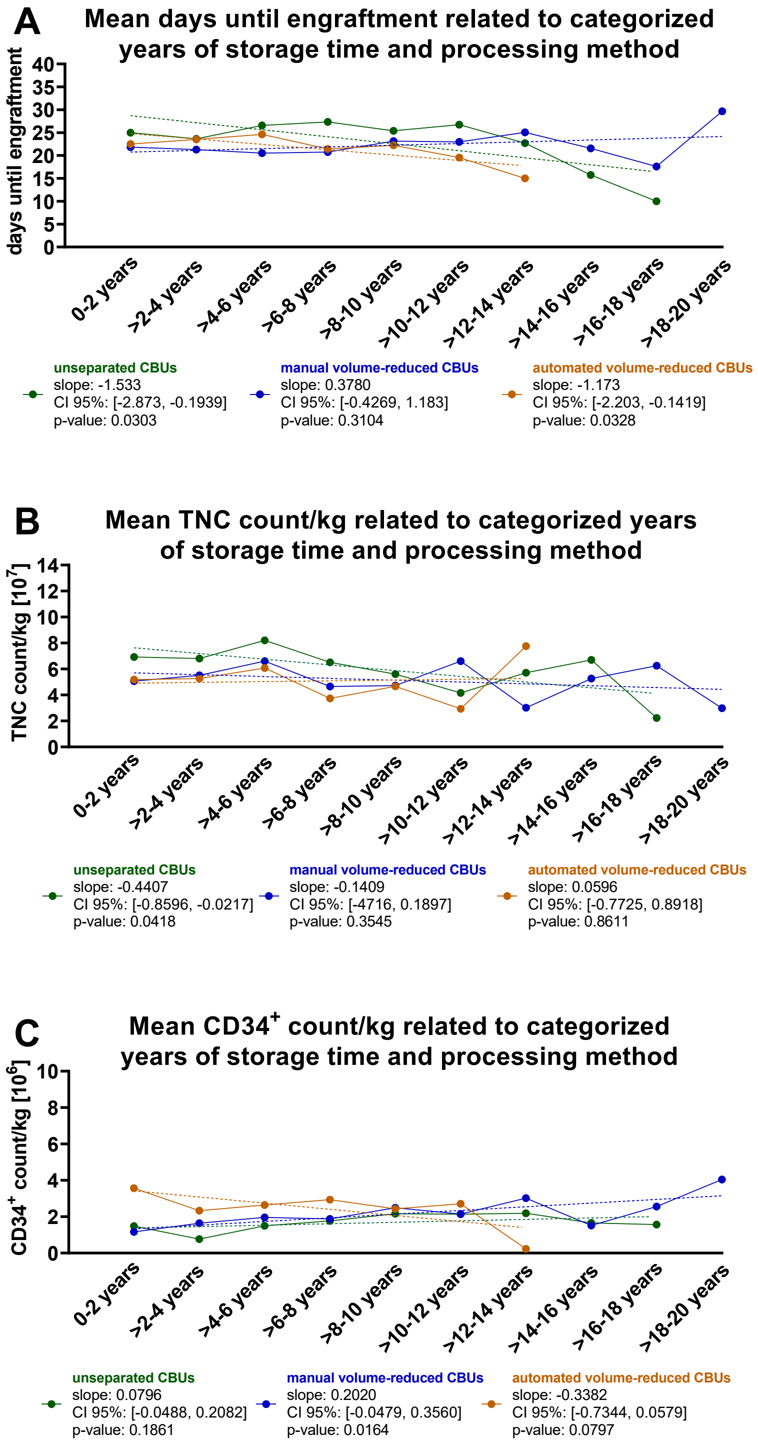

Supplementary Figure 3: Stability outcomes of released transplants split by period of storage per type of final product

Storage time was categorized in two-year steps and according mean values are given for (A) engraftment time, (B) TNC count/kg and (C) $CD34^{+}$ count/kg. Results are given separately for three distinct processing methods: unseparated CBUs (green), manual volume-reduced CBUs (blue) and automated volume-reduced CBUs (orange).

Supplementary Table 4A: Associated mean engraftment time values and sample numbers applied for supplementary Figure 3A

Storage time [years]	unseparated CBUs			manual volume-reduced CBUs			manual volume-reduced CBUs		
	Storage time n=	Engraftment [days]	Engraftment n=	Storage time n=	Engraftment [days]	Engraftment n=	Storage time n=	Engraftment [days]	Engraftment n=
0-2 years	48	24,99	34	64	21,84	50	246	22,50	191
>2-4 years	28	23,65	23	96	21,31	70	224	23,51	187
>4-6 years	33	26,58	26	82	20,53	60	145	24,61	125
>6-8 years	18	27,33	15	105	20,76	79	65	21,44	54
>8-10 years	19	25,41	17	48	23,14	35	28	22,24	21
>10-12 years	22	26,75	16	32	22,96	26	17	19,53	15
>12-14 years	12	22,70	10	17	25,06	16	6	15,00	1
>14-16 years	10	15,75	8	12	21,56	9	n.d.	n.d.	n.d.
>16-18 years	2	10,00	2	6	17,60	1	n.d.	n.d.	n.d.
>18-20 years	n.d.	n.d.	n.d.	3	29,67	3	n.d.	n.d.	n.d.

Supplementary Table 4B: Associated mean TNC count/kg values and sample numbers applied for supplementary Figure 3B

Storage time [years]	unseparated CBUs			manual volume-reduced CBUs			automated volume-reduced CBUs		
	Storage time n=	Mean TNC/kg [10^8]	Mean TNC/kg n=	Storage time n=	Mean TNC/kg [10^8]	Mean TNC/kg n=	Storage time n=	Mean TNC/kg [10^8]	Mean TNC/kg n=
0-2 years	48	6,92	48,00	64	5,06	64	246	5,18	228
>2-4 years	28	6,81	28,00	96	5,51	96	224	5,27	198
>4-6 years	33	8,21	33,00	82	6,61	81	145	6,08	122
>6-8 years	18	6,51	18,00	105	4,65	104	65	3,74	38
>8-10 years	19	5,60	19,00	48	4,72	48	28	4,67	11
>10-12 years	22	4,16	20,00	32	6,60	32	17	2,94	2
>12-14 years	12	5,71	12,00	17	3,02	15	6	7,76	1
>14-16 years	10	6,70	10,00	12	5,27	9	n.d.	n.d.	n.d.
>16-18 years	2	2,23	2,00	6	6,25	5	n.d.	n.d.	n.d.
>18-20 years	n.d.	n.d.	n.d.	3	2,98	2	n.d.	n.d.	n.d.

Supplementary Table 4C: Associated mean $CD34^+$ count/kg values and sample numbers applied for supplementary Figure 3C

Storage time [years]	unseparated CBUs			manual volume-reduced CBUs			automated volume-reduced CBUs		
	Storage time n=	Mean CD34/kg [10^6]	Mean CD34/kg n=	Storage time n=	Mean CD34/kg [10^6]	Mean CD34/kg n=	Storage time n=	Mean CD34/kg [10^6]	Mean CD34/kg n=
0-2 years	48	1,48	44,00	64	1,16	61	246	3,57	228
>2-4 years	28	0,77	24,00	96	1,64	96	224	2,33	198
>4-6 years	33	1,50	28,00	82	1,96	80	145	2,64	122
>6-8 years	18	1,77	17,00	105	1,88	103	65	2,94	38
>8-10 years	19	2,16	19,00	48	2,49	48	28	2,43	11
>10-12 years	22	2,14	20,00	32	2,15	32	17	2,71	2
>12-14 years	12	2,19	12,00	17	3,02	15	6	0,23	1
>14-16 years	10	1,66	10,00	12	1,52	9	n.d.	n.d.	n.d.
>16-18 years	2	1,57	2,00	6	2,56	5	n.d.	n.d.	n.d.
>18-20 years	n.d.	n.d.	n.d.	3	4,05	2	n.d.	n.d.	n.d.

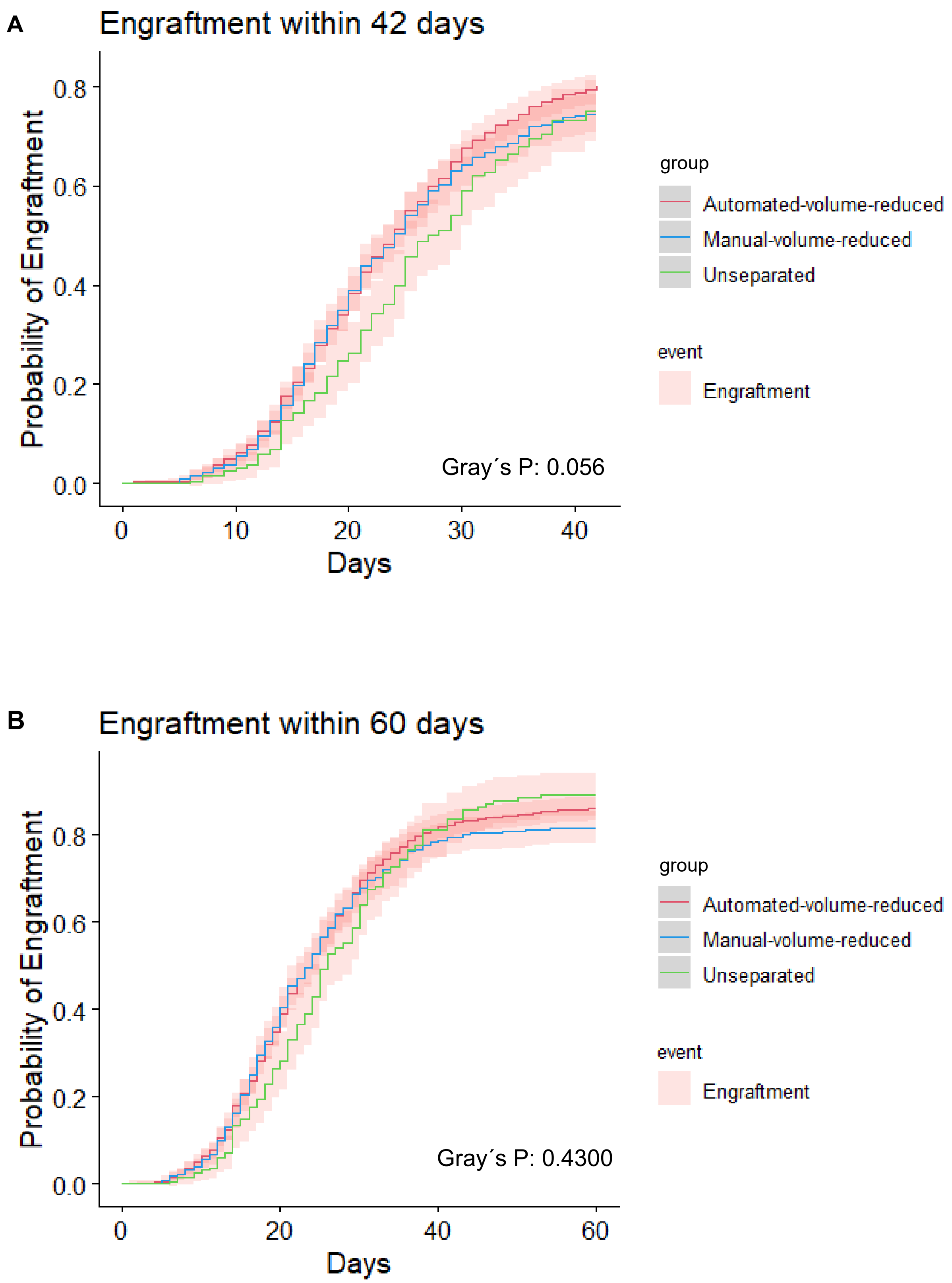

Supplementary Figure 4: Transplant outcomes for distinct processing methods

Cumulative incidence of neutrophil engraftment categorized by distinct processing methods applied. Probabilities of engraftment are assessed after (A) 42 days and (B) 60 days after transplantation categorized for the groups of unseparated CBUs (green), manual volume-reduced CBUs (blue) and manual volume-reduced CBUs (red).

Statistical methods

Statistical analysis for time to event outcomes were performed with the statistical software R, version 4.3.1 (R Core Team, 2023). The cumulative incidence curves were calculated with the R package cmprsk (Gray, 2022) and displayed using the package survminer (Kassambara, et el. 2021). The Gray's statistical significant test implemented in the cmprsk package was used to compare cumulative incidence curves, at a statistical significant level of 5%.

References:

Kassambara A, Kosinski M, Biecek P (2021). _survminer: Drawing Survival Curves using 'ggplot2'_.
R package version 0.4.9,
<https://CRAN.R-project.org/package=survminer>.

Gray B (2022). _cmprsk: Subdistribution Analysis of Competing Risks_. R package version 2.2-11,
<https://CRAN.R-project.org/package=cmprsk>.

R Core Team (2023). _R: A Language and Environment for Statistical Computing_. R Foundation for Statistical Computing, Vienna, Austria.
<https://www.R-project.org/>.

Supplementary Table 5. Cord blood characteristics at transplantation for engraftment vs non-engraftment

Parameter	Engraftment	p-value	Number of values	Minimum	Maximum	Range	Mean	Std. Deviation
TNC Recovery after processing [%]	no	ns 0.8321	121	65,56	108,40	42,81	88,31	7,83
	yes		1143	51,86	127,20	75,29	88,47	7,62
TNC Recovery after thaw [%]	no	ns 0.5229	113	8,69	110,90	102,20	87,81	10,79
	yes		1090	4,23	156,70	152,50	87,01	12,78
TNC count after processing [10^8]	no	ns 0.3483	121	5,84	47,96	42,12	18,31	7,21
	yes		1143	4,91	58,30	53,39	18,88	6,25
TNC count after thaw [10^8]	no	ns 0.4924	113	0,51	38,94	38,43	16,26	6,48
	yes		1093	0,54	59,49	58,95	16,68	6,29
CD34 Recovery after thaw [%]	no	ns 0.8136	83	8,17	426,80	418,60	92,57	54,08
	yes		812	5,00	811,80	806,80	93,90	48,70
$CD34^+$ count after processing [10^6]	no	***0.0448**	88	0,79	29,70	28,91	7,91	6,02
	yes		835	0,61	53,06	52,45	9,41	6,72
$CD34^+$ count after thaw [10^6]	no	ns 0.3447	113	0,06	41,37	41,31	6,96	6,94
	yes		1090	0,04	54,46	54,42	7,57	6,53
TNC Viability after processing [%]	no	ns 0.8470	39	92	99	7	96,72	1,72
	yes		502	85	99	14	96,65	2,17
TNC Viability after thaw [%]	no	ns 0.9690	53	60	98	38	89,83	8,24
	yes		600	40	99	59	89,87	7,70
$CD34^+7AAD^-$ Viability after processing [%]	no	ns 0.2771	10	94	100	6	97,90	2,08
	yes		131	80	100	20	96,62	3,66
$CD34^+7AAD^-$ Viability after thaw [%]	no	ns 0.7928	22	86	99	13	93,23	4,16
	yes		249	73	100	27	92,90	5,64
$CD45^+7AAD^-$ Viability after processing [%]	no	ns 0.9952	10	88	100	12	93,60	4,06
	yes		131	65	100	35	93,59	6,30
$CD45^+7AAD^-$ Viability after thaw [%]	no	ns 0.3850	22	52	88	36	71,32	11,04
	yes		249	45	90	45	69,43	9,64
CFC count after processing [10^6]	no	ns 0.1630	86	0,44	14,26	13,82	2,55	2,30
	yes		834	0,06	17,91	17,85	2,93	2,40
CFC count after thaw [10^6]	no	ns 0.1738	108	0,19	6,16	5,97	2,10	1,34
	yes		1034	0,10	9,18	9,08	2,30	1,46
ClonE (CFC thawed vs $CD34^+$ after processing) [%]	no	ns 0.1992	78	6,29	174,60	168,30	42,26	30,98
	yes		743	1,58	444,30	442,80	37,80	28,97
TNC per kg [10^7]	no	ns 0.7164	133	0,90	47,50	46,60	5,41	6,60
	yes		1148	0,40	81,40	81,00	5,63	6,37
